# Temporal gene profiling of the 5XFAD transgenic mouse model highlights the importance of microglial activation in Alzheimer’s disease

**DOI:** 10.1186/1750-1326-9-33

**Published:** 2014-09-11

**Authors:** Véréna Landel, Kévin Baranger, Isabelle Virard, Béatrice Loriod, Michel Khrestchatisky, Santiago Rivera, Philippe Benech, François Féron

**Affiliations:** 1 Aix Marseille Université, CNRS, NICN UMR 7259, 13916 Marseille, France; 2 APHM, Hôpitaux de la Timone, Service de Neurologie et Neuropsychologie, 13385 Marseille, France; 3 Aix Marseille Université, TAGC UMR 1090, 13288 Marseille, France; 4 INSERM, TAGC UMR 1090, 13288 Marseille, France

**Keywords:** Transcriptome, Hippocampus, Neocortex, Neuro-immune processes, Inflammation, Interferon, Oxidative stress, GTPase signaling, Microglia, Phagocytosis

## Abstract

**Background:**

The 5XFAD early onset mouse model of Alzheimer’s disease (AD) is gaining momentum. Behavioral, electrophysiological and anatomical studies have identified age-dependent alterations that can be reminiscent of human AD. However, transcriptional changes during disease progression have not yet been investigated. To this end, we carried out a transcriptomic analysis on RNAs from the neocortex and the hippocampus of 5XFAD female mice at the ages of one, four, six and nine months (M1, M4, M6, M9).

**Results:**

Our results show a clear shift in gene expression patterns between M1 and M4. At M1, 5XFAD animals exhibit region-specific variations in gene expression patterns whereas M4 to M9 mice share a larger proportion of differentially expressed genes (DEGs) that are common to both regions. Analysis of DEGs from M4 to M9 underlines the predominance of inflammatory and immune processes in this AD mouse model. The rise in inflammation, sustained by the overexpression of genes from the complement and integrin families, is accompanied by an increased expression of transcripts involved in the NADPH oxidase complex, phagocytic processes and IFN-γ related pathways.

**Conclusions:**

Overall, our data suggest that, from M4 to M9, sustained microglial activation becomes the predominant feature and point out that both detrimental and neuroprotective mechanisms appear to be at play in this model. Furthermore, our study identifies a number of genes already known to be altered in human AD, thus confirming the use of the 5XFAD strain as a valid model for understanding AD pathogenesis and for screening potential therapeutic molecules.

## Background

Significant progress has been made uncovering the role of specific genes in Alzheimer’s disease (AD), yet little is known about the global molecular changes leading to neurodegeneration and brain dysfunction. One drawback comes from the fact that brain tissue from AD patients only becomes available *post mortem*, *i.e.* at very late stages of the disease. For this reason, transgenic AD mouse models are precious tools to gain insight into the spatio-temporal changes that may affect molecular cascades involved in disease progression.

The 5XFAD mouse model used in this study bears five mutations linked to familial forms of AD and recapitulates in a few months the main features of AD [1]. All these mutations act in an additive manner to boost the production of β-amyloid (Aβ) peptides, resulting from the processing of amyloid precursor protein (APP), in particular the 42 amino acid form, Aβ42 [2-6]. Compared with other models, 5XFAD mice display AD features much earlier. Though they do not present a clear tau pathology, they develop cerebral amyloid plaques and gliosis as early as 2 months of age [1]. Electrophysiological studies detected hippocampal synaptic dysfunctions in M6 5XFAD animals, concomitant with synaptic loss and memory deficits [7-22]. Progressive neuronal death has been described from M9 onwards in cortical layer 5 neurons and subiculum of 5XFAD mice [12,23], a characteristic that is absent in most AD mouse models.

How these pathophysiological alterations correlate with global spatio-temporal changes in gene expression remains to be thoroughly evaluated. Few prior transcriptomic studies examined AD mouse models, usually at a single time point or in a single brain region [24-29]. Only two studies investigated the transcriptome of 5XFAD mice, one using RNA-seq in frontal cortex and cerebellum of 7 week-old transgenic mice [30], the other using whole-brain next-generation sequencing to compare young (M3-6) versus old (M12) mice from 5XFAD and Tg4-42 strains [31].

Here, we carried out a longitudinal transcriptomic study on two major brain regions affected in AD, the hippocampus and the neocortex, obtained from 5XFAD female mice at presymptomatic (M1), prodromal-like (M4) and symptomatic stages (M6 and M9) of the pathology. We investigated how genes with a modulated expression are involved in functional networks through the use of two text-mining based softwares (Ingenuity and PredictSearch). Among the genes involved in these networks, a bibliographic search was performed to identify those reported in AD patients.

Our results indicate a tremendous shift in the transcriptional profile between M1 and M4 in both the cortex and hippocampus of 5XFAD mice, mainly characterized by an increase in inflammatory and immune markers. Moreover, they emphasize the predominant activation of microglia and transcriptional activities induced by interferon-γ (IFN-γ), likely through the expression of interferon regulatory factor 8 (IRF8), which stands out as a key transcriptional regulator in our study. The main IRF8 target pathways include antigen processing, antigen presentation and phagosome maturation, associated with a modulation of GTPase signaling. Interestingly, a high number of dysregulated genes are associated to AD, confirming that the 5XFAD model mirrors, at an early age, many aspects of this neurodegenerative disease.

## Results and discussion

### Temporal distribution of dysregulated genes reveals dramatic changes from M4 onwards

**Figure 1:**
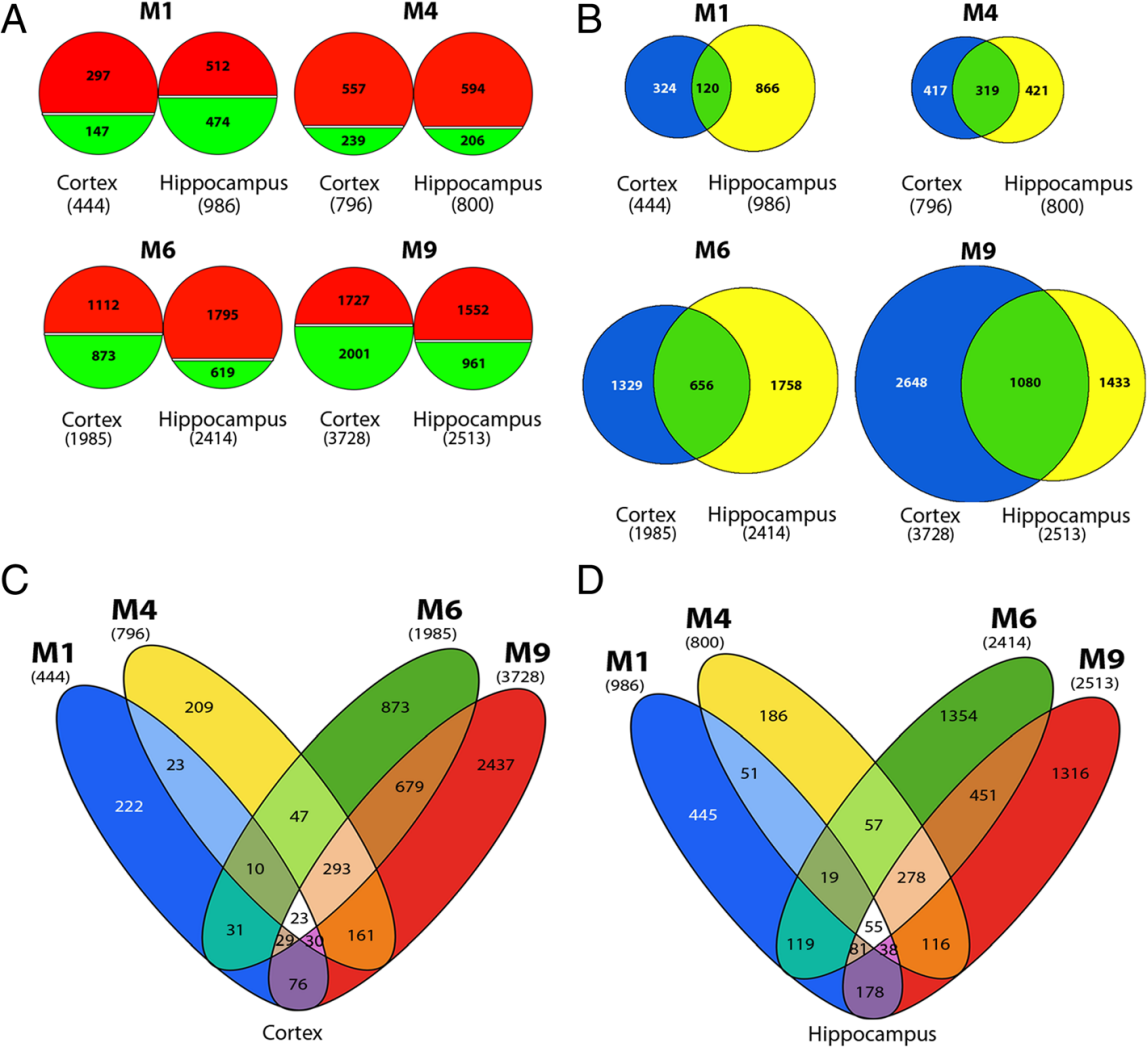
**Overview of gene expression profiles in cortex and hippocampus of 5XFAD mice, at 4 different ages, reveals a shift in expression patterns between M1 and M4. (A)** Number of up- (red) and down- (green) regulated genes in cortex and hippocampus of 5XFAD mice compared with wild type animals at M1, M4, M6 and M9. Total number of DEGs for each brain region at each age is reported in brackets. **(B)** Number of overlapping and non-overlapping DEGs in cortex (blue) and hippocampus (yellow) at M1, M4, M6 and M9. The center of the Venn diagram (green) illustrates the proportion of shared DEGs between both brain regions. **(C and D)** Number of shared and specific DEGs across all ages in cortex **(C)** and hippocampus **(D)**. Analysis was based on the total number of DEGs, both up- and down-regulated at each age. Fold change (FC) cut-off used for above analyses was −1.5 > FC > 1.5 when comparing signals from 5XFAD mice with wild type controls.

Figure 1 summarizes the global screening of gene expression analysis of cortex and hippocampus from 5XFAD compared with wild type mice at M1, M4, M6 and M9. The number of differentially expressed genes (DEGs) increases with age in both tissues (Figure 1A) with a drastic increase between M4 and M6 when considering the number of up- and down-regulated genes (Figure 1A and B).

At M1, twice as many genes are dysregulated in the hippocampus as in the cortex (Figure 1A), suggesting that distinct alterations occur in these two regions at that early stage, as described at the histological level [1]. Additionally, we observed many shared DEGs between cortex and hippocampus (Figure 1B), in particular at M4, when nearly 50% of the DEGs are common to both brain regions. By contrast, at M1, only 12% of the genes modulated in hippocampus overlap with those found in cortex (Figure 1B).

In order to evaluate the specificities at each studied stage, we looked at overlapping and non-overlapping DEGs in transgenic animals, from M1 to M9, separately in cortex and hippocampus. Interestingly, the expression of only 23 genes from the cortex and 55 from the hippocampus (Figure 1C and D, white areas) is modulated across all ages (Additional file 1: Table S1). Strikingly, the expression of an important number of genes (293 for the cortex; 278 for the hippocampus; Figure 1C and D, light orange areas) is altered through M4 to M9. Among those, 183 DEGs at M4, M6 and M9 are common to both tissues (Additional file 2: Table S2). Most of these genes are upregulated; only 3 of these DEGs show decreased expression at all three ages and in both regions, while 176 display an increased expression and 4 are inconsistently either up- or down-regulated in cortex or hippocampus (Additional file 2: Table S2).

### DEG-related functions support alterations in inflammation pathways and behavior

**Figure 2:**
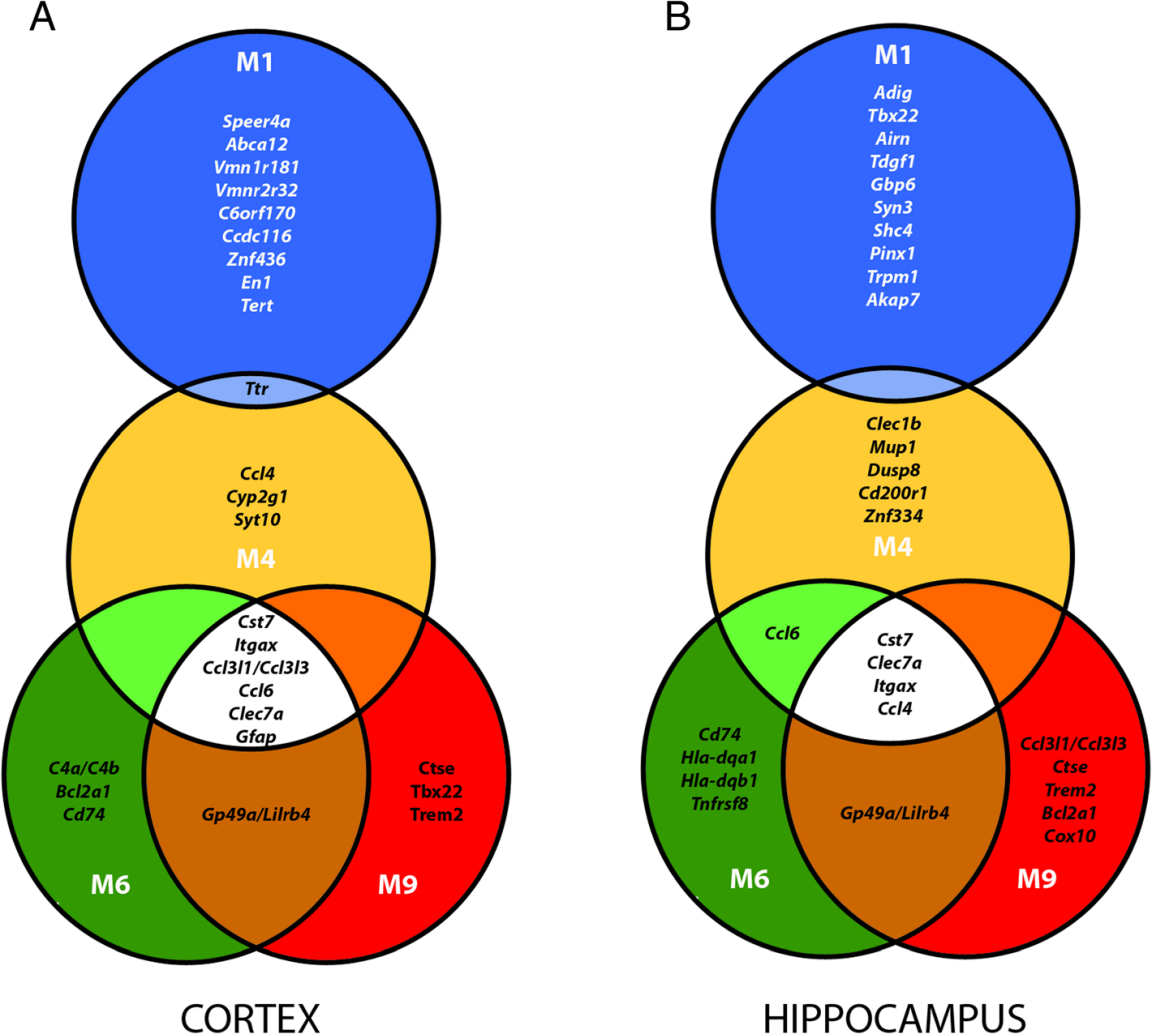
**The top ten most up-regulated genes at each age, in cortex and hippocampus, illustrate the alteration of inflammatory and immune processes from M4 onwards.** Venn diagrams representing the top ten up-regulated genes in cortex **(A)** and hippocampus **(B)** of 5XFAD mice at M1 (blue), M4 (yellow), M6 (green) and M9 (red). Genes are listed by rank of fold change, the cut-off for analysis being FC > 1.5 when comparing gene probe signals from 5XFAD with wild type mice. Note that only *Ttr* dysregulation is present at both M1 and M4 in the cortex while the other 9 genes are specific to M1. However, at M4, M6 and M9, 60% and 40% of these genes are consistently dysregulated in the cortex and the hippocampus, respectively.

To investigate whether these quantitative changes might reflect alterations of specific processes and/or pathways contributing to disease progression, we looked at the most upregulated genes in both tissues over time (Figure 2) using Ingenuity Pathway Analysis (IPA). Six out of the top ten upregulated genes from the cortex and four from the hippocampus at M4 also appear among the top ten upregulated genes at M6 and M9 (Figure 2). Inflammation and immunomodulation are the main affected processes in these tissues, as illustrated by changes in expression of *Clec7a* (coding for the dectin-1 protein), *Cst7* (cystatin F), *Itgax* (Cd11c) and genes encoding chemokines Ccl3, Ccl4, Ccl6 and the glial fibrillary acidic protein (Gfap) (Table 1).

**Table 1:** Upregulated genes with their fold change related to inflammation and immune processes in the cortex and hippocampus of 5XFAD mice at M1, M4, M6 and M9

		**Cortex**				**Hippocampus**			
**Gene**	**Probe**	**M1**	**M4**	**M6**	**M9**	**M1**	**M4**	**M6**	**M9**
*Clec7a*	A51P246653	0.8	**12.1**	**35.0**	**30.9**	1.1	**15.9**	**32.2**	**32.5**
*Cst7*	A51P137419	1.0	**74.7**	**63.5**	**140.2**	1.3	**100.5**	**118.4**	**145.8**
*Itgax*	A51P303424	0.7	**16.3**	**13.4**	**19.6**	0.8	**16.1**	**14.8**	**23.2**
*Ccl3*	A51P140710	1.0	**15.8**	**14.7**	**15.4**	0.6	**11.3**	**7.3**	**17.3**
*Ccl6*	A51P460954	1.4	**12.4**	**9.4**	**13.4**	1.4	**13.7**	**10.8**	**9.7**
*Gfap*	A55P2157245	1.1	**3.4**	**6.1**	**4.4**	1.1	**2.2**	**3.0**	**5.4**
*Gfap*	A52P52303	1.1	**5.2**	**8.4**	**5.0**	1.1	**2.8**	**3.1**	**4.5**
*Gfap*	A55P2157250	1.2	**8.1**	**8.6**	**11.8**	0.9	**4.0**	**8.0**	**3.5**
*Ccl4*	A51P509573	0.9	**9.0**	**7.3**	**9.9**	0.9	**12.7**	**10.7**	**11.3**
*Ttr*	A65P19832	**12.2**	**8.7**	**2.5**	0.7	0.2	1.2	1.0	**1.5**
*Kl*	A52P439358	**2.9**	**3.0**	1.2	0.5	0.2	1.2	0.6	**2.2**

Fold changes are indicated in bold when FC > 1.5.

**Figure 3:**
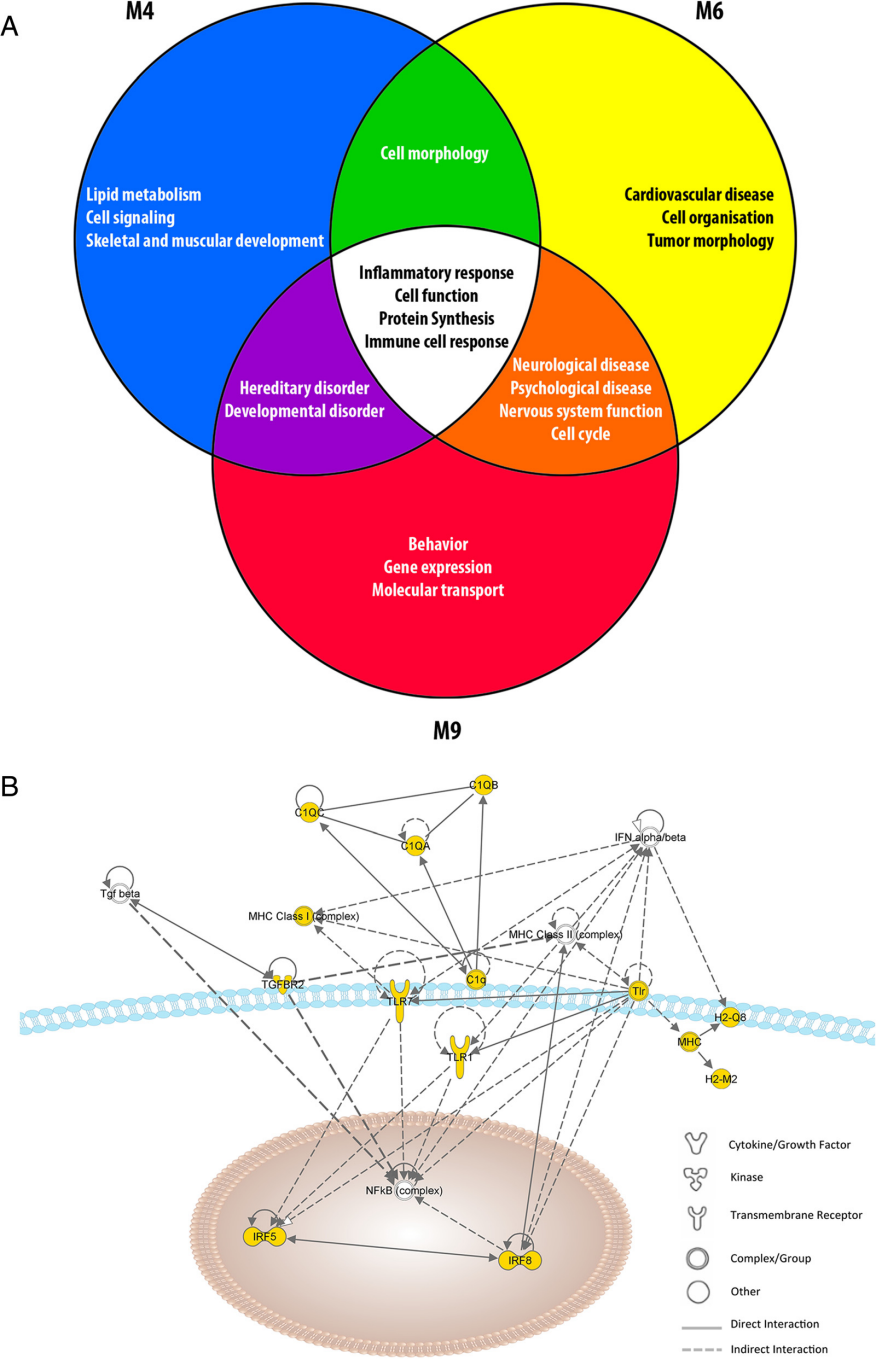
**Metabolic pathways associated to gene dysregulation in 5XFAD cortex and hippocampus at M4, M6 and M9: common versus age-specific processes.** Biological functions and metabolic pathways associated to gene expression dysregulation were identified using Ingenuity Pathway Analysis (IPA). **(A)** Data from both the cortex and hippocampus were analyzed as one dataset and the main metabolic pathways affected were clustered according to age (M4, blue; M6, yellow; M9, red). The metabolic pathways affected across all three ages are represented in white and are related to inflammatory and immune responses. **(B)** The main metabolic pathway affected at M4, M6 and M9 relates to infectious disease, cellular function and antigen presentation.

In contrast to these overlaps, the top ten upregulated genes in M1 animals are fundamentally distinct from those found in older animals (Figure 2). Only the transthyretin (*Ttr*) gene, encoding a transporter of thyroxin and retinol, is strongly upregulated in the cortex at both M1 and M4 (Figure 2). Ttr, previously identified as upregulated in the frontal cortex of young, presymptomatic 5XFAD mice [30,31], is able to bind and sequester Aβ peptide, thereby preventing its aggregation and plaque formation [32-34]. The finding that high levels of *Ttr* precede plaque deposition is corroborated by another study performed on Tg2576 transgenic mice, a model in which plaque deposition does not occur until M12 [35]. Moreover, *Ttr* was identified as a physiological target of APP, since its expression is increased by soluble extracellular APP processed products [34]. Interestingly, *Klotho* (*Kl*), encoding a hormone involved in aging processes such as oxidative stress and calcium homeostasis [36-38], was similarly dysregulated. Both genes exhibit a similar expression profile in our transcriptomic study (Table 1), confirming that *Ttr* and *Kl* are co-regulated APP targets. In the cortex, their expression decreases progressively from M4 to M6 to reach, at M9, expression levels below those of wild type mice. Conversely, in the hippocampus of 5XFAD mice, their expression is repressed at M1 and upregulated at M9. Such differential expression patterns may highlight distinct temporally-regulated protective capacities, as observed in cultures of oxygen- and glucose-deprived astrocytes from cortex and hippocampus [39].IPA was then undertaken to investigate the most affected networks during the time course of disease progression. When considering all DEGs at M4, M6 and M9 (after the onset), processes associated to the dysregulated genes mainly relate to inflammatory and immune responses (Figure 3A). As an example, Figure 3B maps some genes associated to the complement, major histocompatibility complexes (MHCs) and toll-like receptors (TLRs).

Such a presence of immune markers in the hippocampus and cortex of the 5XFAD mice is not surprising since they are produced in neurons, astrocytes and resident microglia. In a healthy brain, they illustrate the “neuro-immune” system that exists in the central nervous system, where, for instance, complement cascade tags neurons destined to destruction [40]. With age and repeated insults, these processes may spiral out of control and lead to degeneration. Through the upregulation of inflammatory/immune markers, the 5XFAD brain recapitulates the dysfunction of the resident immune network seen in AD [41,42].

In addition to inflammatory and immune changes, IPA identified functional links between genes affected from M6 onwards and neurological and psychological diseases (Figure 3A). Changes in genes associated with impaired cognitive functions are found to be a significant feature of M9. This is consistent with published studies reporting abnormal behaviors in 5XFAD mice starting at M6 and strongly consolidated by M9 [7,12,13,19,20,43,44].

These observations suggest that disrupting the intricate balance between neurons and surrounding immune cells may lead to neuronal dysfunction and cognitive deterioration [45,46].

### Establishment of neuroinflammation through activation of complement

A dramatic increase in inflammation stands out as the most striking transcriptomic result. Neuroinflammation is a well-known hallmark of AD and is characterized by the activation of astrocytes and microglia, which appears in the 5XFAD mouse model near and concomitantly to amyloid plaques [1]. In various types of brain insults, peripheral leukocytes infiltrate the injured brain [47-49] and intensify the neuroinflammatory response through pro-inflammatory mediators, free radicals, lipid peroxidation and oxidative stress [50-52]. Their infiltration is mediated, in part, by CD11/CD18 integrins expressed in neutrophils and monocytes/macrophages.

In our study, in addition to Cd11c-encoding gene *Itgax*, genes such as *Itgam*, *Itgb3*, and *Itgb2*, encoding respectively Cd11, Cd61 and Cd18, are all overexpressed in the 5XFAD mice (Table 2). Most of these integrins are transcriptionally induced by IL-1β and/or Aβ through TLR2-mediated signaling [53]. In keeping with this, we found an upregulated expression of *Tlr2* at M4, M6 and M9 (Table 2). Increases in transcript levels of inflammatory markers such as *Cd11b*, *Il-1*β, *Tnf-α* and *Tlr2* have already been observed in 6 months old 5XFAD mice [54].

**Table 2:** Upregulated genes related to complement activation in the cortex and hippocampus of 5XFAD mice at M1, M4, M6 and M9

		**Cortex**				**Hippocampus**			
**Gene**	**Probe**	**M1**	**M4**	**M6**	**M9**	**M1**	**M4**	**M6**	**M9**
*Itgam*	A55P1977929	0.9	**1.5**	1.2	**1.9**	1.1	**2.0**	**1.8**	**1.6**
*Itgb2*	A51P262208	1.0	**2.5**	**2.6**	**3.4**	1.0	**2.3**	**2.4**	**2.5**
*Itgb3*	A52P553890	0.9	1.3	**2.0**	**1.5**	0.9	**1.8**	**1.9**	**3.0**
*Tlr2*	A51P452629	1.0	**2.7**	**3.6**	**3.3**	0.6	**3.2**	**3.6**	**5.1**
*C3*	A51P110301	**1.6**	1.0	**3.5**	**1.6**	0.9	1.2	**5.2**	**6.3**
*C4b*	A55P2078633	1.1	**2.6**	**9.0**	**3.7**	0.8	**2.6**	**4.0**	**5.6**
*C1qa*	A51P181451	1.1	**2.6**	**3.2**	**4.0**	1.0	**3.0**	**3.8**	**4.7**
*C1qb*	A51P351860	1.0	**2.3**	**2.9**	**3.2**	1.0	**2.6**	**5.1**	**2.9**
*C1qc*	A51P102789	1.0	**2.8**	**2.0**	**5.7**	1.2	**3.0**	**4.6**	**4.2**

Fold changes are indicated in bold when FC > 1.5.

**Figure 4:**
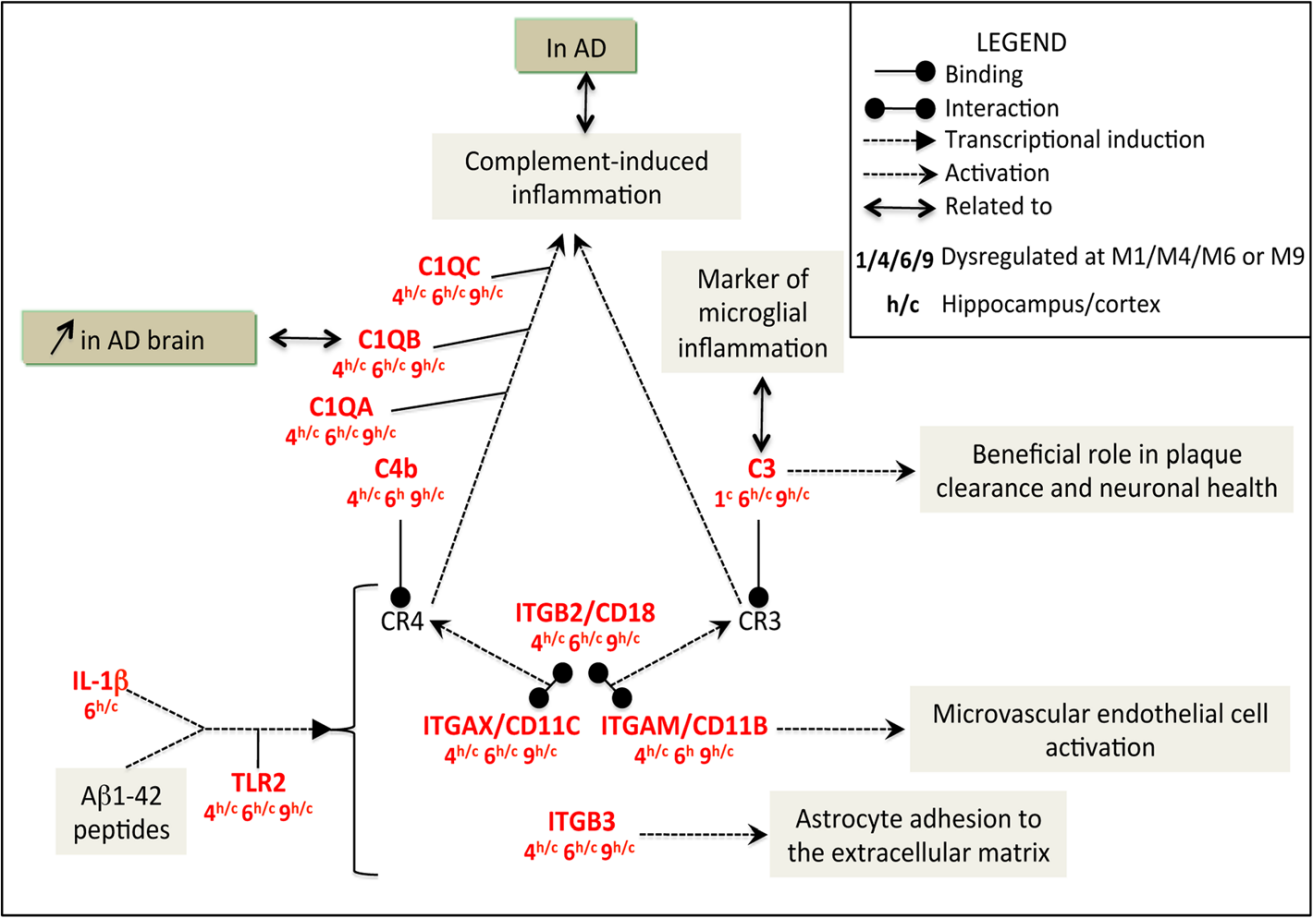
**The complement-induced inflammation pathway: an important mediator of neuroinflammatory processes from M4 onwards.** This functional network was designed using the text-mining software, PredictSearch, based on more stringent criteria than for the above figure: only upregulated DEGs (with a FC > 1.5) found in both the cortex and hippocampus and at consecutive time points (M4/M6; M6/M9 or M4/M6/M9) were considered for analysis. Top right corner: legend for Figures 4, 5, 6, 7.

Moreover, the expression of several members of the complement, known immune effectors, is also upregulated, such as *C3, C4*, *C1qa*, *C1qc* and *C1qb*, which are all overexpressed in the cortex of AD patients [55]. CD18 interacts with CD11b or CD11c to form the C3 receptor (CR3) and C4 receptor (CR4), respectively. C3 and C4 ligands bind to their cognate receptors, C3R and C4R (Figure 4). Activation of these receptors is reportedly part of the complement-induced inflammation in AD mouse models and patients [56-61] and influences microglia to adopt protective or deleterious phenotypes in AD [31,62].

On the whole, our results point to a major raise in inflammation in the 5XFAD brain, linked to complement activation and presumably to immune cell infiltration, reminiscent of similar findings in AD patients and other mouse models [63-68].

### Altered expression of interferon gamma-induced genes

**Figure 5:**
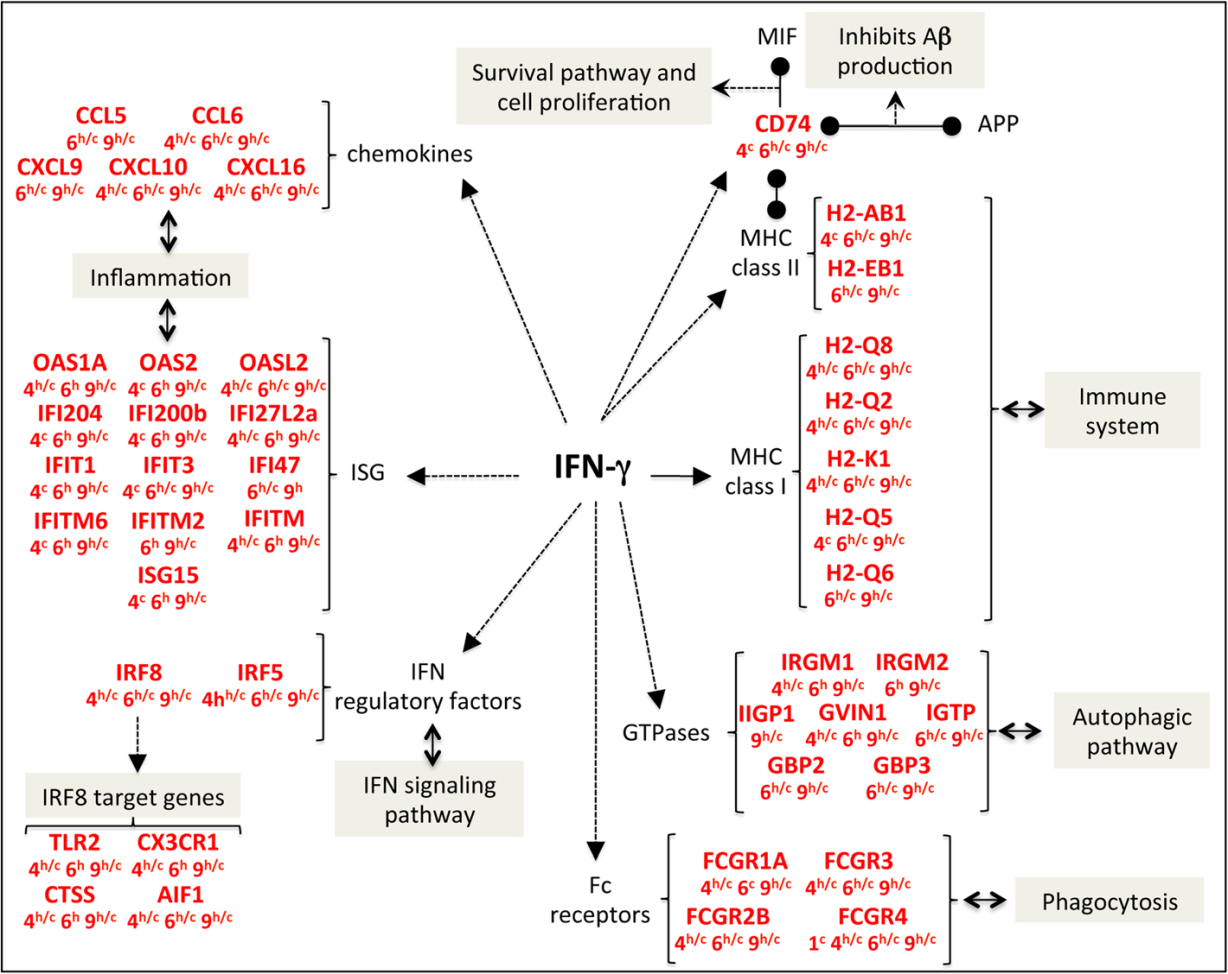
**A large proportion of upregulated genes belong to the family of IFNγ-induced genes: engagement of inflammatory, immune, autophagic and phagocytic pathways.** Legend for this figure is located in the top right corner of Figure 4.

In a second approach, we applied more stringent criteria for gene selection (see Materials and methods) to identify functional networks highly modulated in our model: we focused on genes upregulated in both hippocampus and cortex, from M4 to M9 or at consecutive time points (M4/M6 and M6/M9). We then explored the published data on these dysregulated genes to map out the molecular and cellular players at stake. To this end, we used IPA as well as PredictSearch, another software for the design of functional networks [69-74]. This combined analysis reveals numerous IFN-γ-induced genes, which belong to a larger set of genes known as interferon stimulated genes or ISGs (Figure 5). However, we did not observe any change in IFN-γ gene expression in our study. Although post-transcriptional regulation cannot be excluded, we can postulate that IFN-γ transcription occurs either transiently in brain cells or outside the brain, in peripheral blood cells. IFN-γ can be produced in the brain by glial cells [75]. However, several reports argue in favor of T cell infiltration in AD [63,64]. In *post mortem* AD brain, peripheral T cells cluster around plaques in areas of important gliosis [76-78]. Disruption of the blood-brain barrier has been reported in M8 5XFAD mice [79]. A recent study also demonstrates that a significant infiltration of T cells occurs in the brain of APP/PS1 transgenic mice and that these cells secrete IFN-γ [80]. In the same study, transferring Aβ-specific Th1 cells to APP/PS1 mice increased microglial activation and Aβ deposition and worsened cognitive performances in the Morris water maze. These observations warrant more studies to test the hypothesis that, in the 5XFAD brain, infiltrated T cells may potentially release IFN-γ, activate microglia and stimulate expression of IFN-γ induced genes.As described below, these genes are involved in the regulation of different processes, including immunity, inflammation and GTPase signaling (Figure 5).

Concerning the potential role of the immune system, major histocompatibility complex (MHC) genes are expressed in microglia upon cytokine stimulation in the inflamed brain [81] and both MHC class I and II are known to be upregulated in sporadic forms of human AD [82]. A role for MHC class II genes in the 5XFAD mice is supported by the parallel increase in the expression of *Cd74* (Figure 5), which acts both as chaperone for MHC class II molecules and as receptor for MIF (macrophage migration inhibitory factor). CD74 is also increased in AD cases compared with age-matched controls, notably in neurofibrillary tangles-bearing neurons, amyloid plaques and microglia [83]. Thus, expression of MHC class II genes highlight the predominance of microglia activation in the 5XFAD mice. In response to IFN-γ, a rise in microglial MHC class II genes may enhance antigen presentation to T cells, which, in turn, might contribute to immune-mediated damages to neurons. Interestingly, at least at a certain stage of the disease, *Cd74* expression may also denote a protective effect. Indeed, interaction of CD74 with MIF promotes a cell survival pathway and its interaction with APP blocks Aβ production [84,85].

In our analysis, a second large cluster of genes induced by IFN-γ and dysregulated in 5XFAD brain encodes proteins with GTPase activity (Figure 5), such as Irgm1 and Irgm2 (members of the Immunity-Related GTPase family M [86]), Igtp/Irgm3 (IFN-γ induced GTPase), and Iigp1/Irga6 (interferon induced GTPase 1). Moreover, the expression of another GTPase gene, *Gvin1* (very large interferon inducible 1) and two members of the guanylate-binding protein genes, *Gbp2* and *Gbp3*, is also higher in 5XFAD animals. GBPs belong to another family of GTPases and are the most abundant proteins that accumulate in fibroblasts or macrophages in response to IFN-γ stimulation [87]. A number of other GTPases, not described to be regulated by IFN-γ, but related to TGF-β activity, are also overexpressed in our model.

GTPases such as Irgm1 regulate, during ischemic stroke, survival and neuronal autophagy [88], a process that eliminates dysfunctional cell components using lysosomes. Irgm1 exacerbates experimental auto-immune encephalomyelitis by promoting disruption of the blood-brain and blood-cerebrospinal fluid barriers [89]. In addition to controling cell death or survival [90-92], murine IRGs affect protein aggregate formation and clearance [93,94] and altogether, these functions engage the autophagic pathway [91].

A possible involvement of autophagy in 5XFAD animals is also supported by the increased expression of known targets of Tfeb, a transcriptional factor involved in lysosomal biogenesis [95,96]. These Tfeb targets include *Gusb* (beta-glucuronidase), *Naglu*, *Hexa*, *Hexb*, and *Ctsd*[95,97], which are greatly expressed in the 5XFAD mice from M4 to M9 in both cortex and hippocampus (Table 3). Interestingly, the expression of *Gusb* was upregulated in Aβ-resistant cells and might illustrate a protective effect against Aβ toxicity [98]. Moreover, a role for presenilins in regulating lysosomal function has been demonstrated [97] and several lines of evidence suggest that endosomes/lysosomes are involved in Aβ production [99,100]. Thus, our results highlight the possible activation of the endosomal/lysosomal system in the 5XFAD brain, in keeping with observations in this model [101] and in human AD brains [102].

**Table 3:** Upregulated genes, fold changes, and associated functional categories for genes consistently dysregulated from M4 to M9

		**Cortex**				**Hippocampus**				
**Gene**	**Probe**	**M1**	**M4**	**M6**	**M9**	**M1**	**M4**	**M6**	**M9**	
*Gusb*	A51P211491	0.9	**2.5**	**3.2**	**3.2**	1.1	**3.0**	**3.6**	**3.9**	**Lysosomal biogenesis**
*Naglu*	A52P504361	1.0	**2.1**	**2.1**	**2.2**	0.8	**1.7**	**2.2**	**2.0**	
*Hexa*	A51P282667	1.1	**1.9**	**2.0**	**2.3**	1.2	**1.7**	**2.3**	**2.4**	
*Hexb*	A51P453111	1.0	**2.5**	**2.0**	**3.2**	0.9	**2.8**	**2.0**	**3.3**	
*Ctsd*	A65P13209	1.1	**3.0**	**4.4**	**2.4**	0.9	**2.8**	**3.8**	**3.1**	
*Tgfb1*	A51P390715	1.1	**1.8**	**1.7**	**2.3**	0.8	**2.3**	**2.6**	**1.6**	**TGF-β signaling**
*Tgfbr1*	A51P2137206	1.1	**1.5**	**1.7**	**1.7**	0.8	**1.6**	1.4	**1.9**	
*Tgfbr2*	A51P450573	1.0	**1.9**	**2.1**	**2.3**	1.0	**2.2**	**2.0**	**3.7**	
*Aif1*	A51P400543	1.0	**2.1**	**2.4**	**2.2**	1.0	**2.5**	**2.3**	**2.9**	**Markers of microglial activation**
*Ptprc*	A55P1990324	1.0	**2.0**	**1.7**	**2.0**	1.0	**2.0**	**2.1**	**2.5**	
*Cd86*	A55P1971951	0.9	**1.8**	**2.3**	**2.5**	1.1	**2.5**	**3.0**	**3.5**	
*Cd14*	A51P172853	1.0	**2.7**	**3.2**	**3.8**	1.0	**2.6**	**3.6**	**3.3**	
*Igf1*	A55P2031631	0.9	**2.6**	**2.0**	**5.1**	1.0	**3.0**	**3.0**	**4.2**	**Protective activities**
*Osmr*	A51P319460	0.9	**1.6**	**2.7**	**3.1**	0.7	**2.5**	**3.0**	**5.3**	
*Grn*	A51P192800	0.9	**2.5**	**2.3**	**2.6**	1.1	**2.2**	**3.0**	**2.5**	

Fold changes are indicated in bold when FC > 1.5.

### Potential role of IRF8 in the expression of interferon gamma-regulated genes

We next explored in more detail which pathways were activated immediately downstream of IFN-γ in the 5XFAD brain. As detailed below, we discovered an unexpected downstream effector of IFN-γ: Interferon Regulatory Factor 8 (IRF8). Induction of MHC class I genes by IFN-γ generally depends on the JAK/STAT pathway that targets the ISRE (Interferon Stimulating Response Element) motif [103]. By contrast, the expression of MHC class II genes requires that the transcriptional factor CIITA forms a complex with DNA-binding factors targeting the MHC class II promoter [104]. The increased expression of both MHC class I and II genes observed in 5XFAD mice suggests that the two IFN-γ-signaling pathways are altered. However, among the factors known to participate in these pathways, only *Stat3* and *Socs3* display a significant upregulated expression in both tissues of 5XFAD mice, and not before M9. Therefore, it can be assumed that novel pathways explain the major rise in IFN-γ-induced genes.

Among interferon-stimulated genes, it is noteworthy that genes encoding transcription factors *Irf5* and *Irf8* are highly upregulated in the 5XFAD mice, from M4 to M9 (Figure 5). Irf8 stands out as a particularly interesting effector of IFN-γ. Expressed predominantly in hematopoietic cells and further increased upon treatment with IFN-γ [105-107], Irf8 is required to propagate pro-inflammatory signals and to activate microglia [108]. IRF8 also instructs myeloid progenitors to become mononuclear phagocytes [109,110]. A combination of genome-wide methods already confirmed the crucial role of IRF8 in regulating early immune response, including phagosome maturation, antigen processing and presentation [111,112]. Interestingly, 5XFAD mice overexpress most of the Irf8 target genes supporting these processes (Figure 5). This strongly suggests that Irf8 accounts for the IFN-γ regulated processes described here and therefore represents a key player in the 5XFAD pathology.

Alternatively, IRF8 expression can also be induced through TGF-β [113], which is highly overexpressed in 5XFAD mice, along with transcripts for its receptors, *Tgfbr1* and *Tgfbr2* (Table 3). This pathway inhibits macrophages and suppresses microglial expression of MHC antigens [114,115], and its activation in 5XFAD brain might therefore be a protective response against sustained microglial activation.

### Involvement of NADPH oxidase (NOX) complex in microglial activation

The NOX complex contributes to persistent microglial activation and reactive oxygen species production, which leads to an increase in oxidative stress, regarded as an early sign of AD pathophysiology [116,117]. Moreover, studies on different AD mouse models, including 5XFAD mice, reported increased levels of oxidative damage, which appear before Aβ deposition and therefore constitute an early event in disease pathogenesis [24,44,118,119]. Actually, a recent proteomic analysis revealed that the most affected biological processes in M4 5XFAD hippocampus include cell redox homeostasis and response to oxidative stress [120].

**Figure 6:**
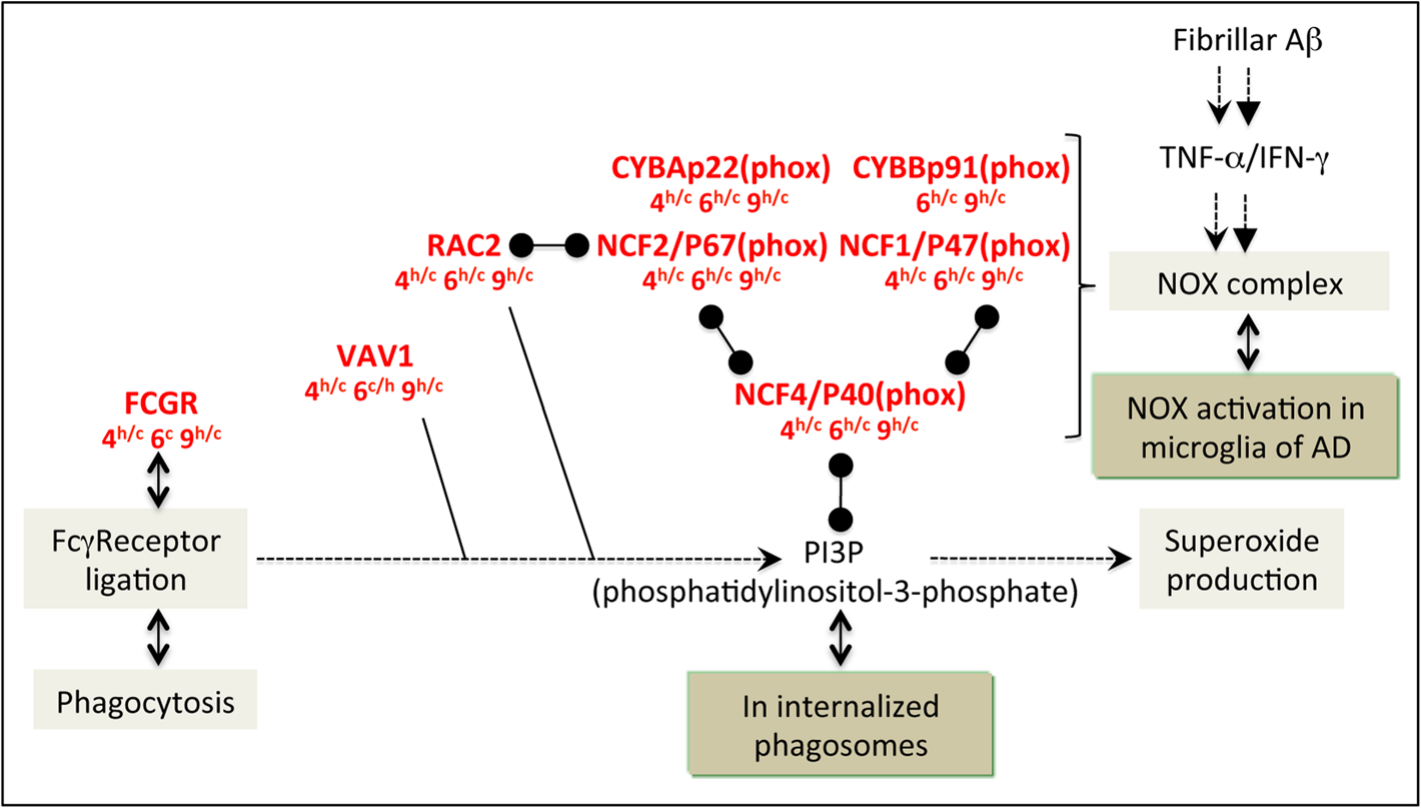
**Increased expression of genes involved in the NADPH oxidase complex.** Legend for this figure is located in the top right corner of Figure 4.

In our study, most of the genes encoding NOX components as well as NOX associated factors, Fc receptors, Vav and Rac2, are overexpressed as early as M4 in 5XFAD mice (Figure 6). Of note, genes encoding the NOX subunits as well as Fcgr1a/CD64 (Fc fragment of IgG receptor, high affinity 1a) are regulated by IFN-γ [107,121-123]. Their co-expression is in line with the observation that clustering of Fcγ receptors activates VAV proteins (Rho/Rac guanine nucleotide exchange factors), leading to robust superoxide generation through NOX [124-126].

Several studies provide strong evidence for the involvement of NOX and its downstream oxidative signaling pathway in the toxic effects elicited by Aβ. In AD brains, Aβ is thought to act on NOX in microglial cells, which produce neurotoxic superoxide [127]. Aβ also induces oxidative stress in hippocampal astrocytes through a mechanism sensitive to NOX inhibitors [128].

According to these observations, NOX activation can occur in either microglia or astrocytes. However, it is likely that, in our model, NOX is predominantly activated in microglia. Indeed, in addition to MHC class II genes and *Cd74*, markers of microglial inflammation are strongly overexpressed in 5XFAD mice (Table 3), notably *Aif1/Iba1*, *Ptprc/Cd45*, *Cd68*, *Cd86* and *Cd14*[83,129-131]. In line with the microglial origin of NOX activation, expression of the Fcγ receptor-encoding genes is found around senile plaques and in ramified microglia, throughout the cortex and the white matter of normal and AD brains [132]. Furthermore, RAC2, which controls NOX activation by preferentially interacting with the NCF2/p67(phox) NOX subunit [133], is largely predominant in human phagocytes [134].

### Microglial phagocytosis

**Figure 7:**
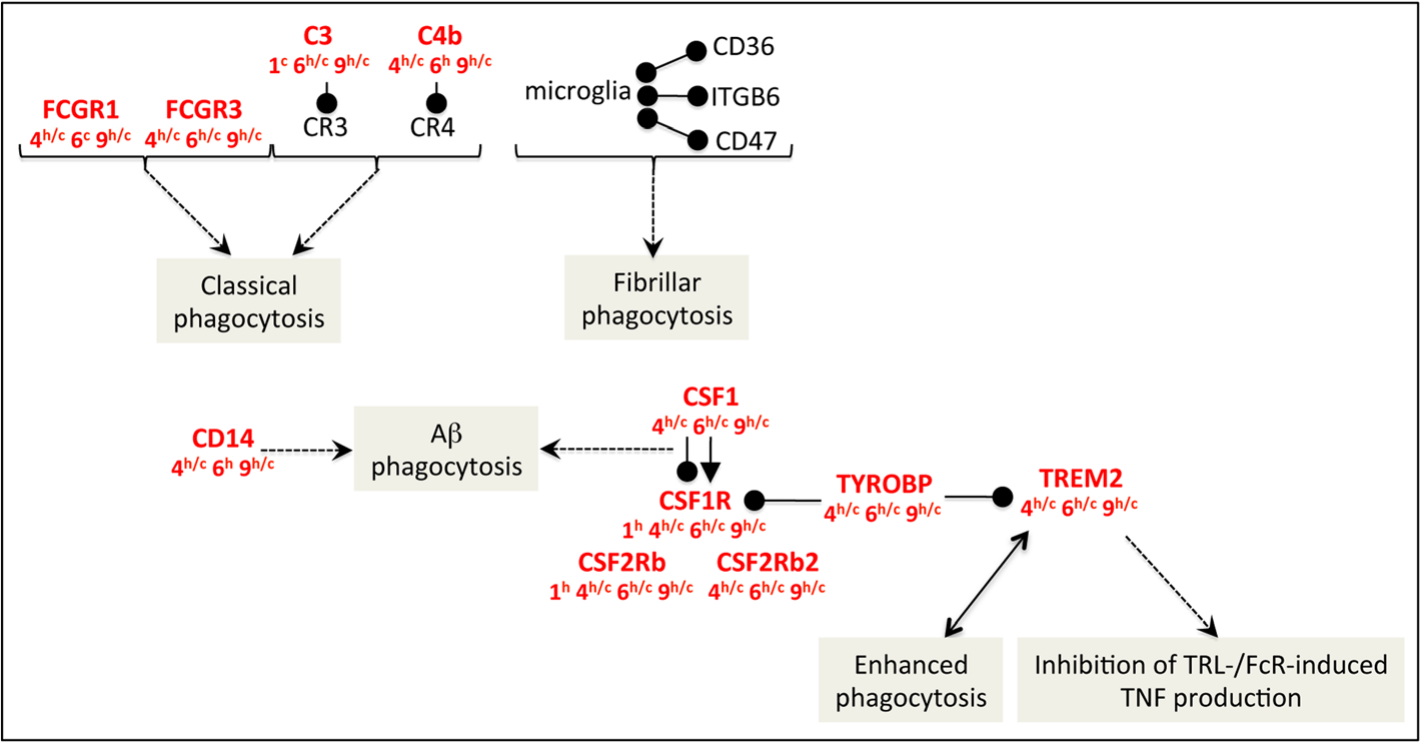
**Increased microglial activation and induction of associated neuroprotective signaling pathways.** Legend for this figure is located in the top right corner of Figure 4.

Microglia/brain macrophages constitute about 12% of the cells in the central nervous system, and, in addition to antigen presentation, exhibit phagocytosis. Microglia can phagocytose Aβ fibrils *in vitro* and *in vivo*[135]. Nevertheless, Aβ phagocytosis is inefficient in AD brain despite the presence of abundant activated microglia [136]. One possible explanation may be that exposure of microglia to fibrillar Aβ *in vitro* can induce mechanisms distinct from those used by classical phagocytic receptors, FCGR1 and FCGR3, or complement receptors (Figure 7). This novel phagocytosis would require the interaction of microglia with CD36, ITGB6, and CD47 [137].

Phagocytosis of Aβ by microglia can be also mediated by CD14 [138], which is detected in brains of AD patients, or by CSF1 (macrophage colony stimulating factor 1) and its receptor, CSF1R (Figure 7). Interestingly, Csf1r signaling in injured neurons facilitates protection and survival [139]. The Csf1/Csf1r complex co-signals through Tyrobp, which, together with its receptor, Trem2, activates signal transduction leading to brain myelination and inflammation [140]. The signaling involving Csf1/Csf1r/Trem2/Tyrobp likely plays a role in the 5XFAD physiopathology since all the corresponding genes are strongly upregulated from M4 to M9 in cortex and hippocampus (Figure 7). Moreover, recent reports show that rare *TREM2* variants predispose to AD [141,142] and *Trem2* mRNA and protein are increased in a transgenic mouse model of AD [141]. It has been proposed that these changes represent a response to rising levels of Aβ [141]. Localized to microglia around plaques and neurons in AD models, Trem2 controls two signaling pathways that regulate the reactive phenotype in microglia (Figure 7). The first of these pathways couples increases in *Trem2* expression on microglia with enhanced phagocytosis [143-145]. This could lead to the removal of cell debris and the clearance of Aβ in AD and promote the alternative “protective” activation state of microglia. The second Trem2 signaling pathway suppresses inflammatory reactivity and represses cytokine production and secretion [144], notably TLR- and FcR-induced TNF production [146,147]. Thus, in addition to its protective role by activating phagocytosis of apoptotic neurons and Aβ, the predominant role of the TYROBP/TREM2 complex might be to inhibit, rather than to activate, the innate immune system.

Altogether, these transcriptional changes indicate that, in 5XFAD mice, microglial activation and the resulting phagocytosis are predominant processes, which are already initiated at M4 and maintained at least until M9.

### Other neuroprotective activities

The 5XFAD transcriptomic data identified numerous genes involved in neuroprotective pathways. In addition to those already discussed, we found an upregulation of genes encoding Igf1 (insulin growth factor 1), Osmr (oncostatin M receptor) and Grn/Pgrn (granulin) (Table 3).

Insulin and IGF1 signaling is disturbed in AD brain and in 5XFAD hippocampus [120,148,149]. Igf1 can either protect or increase LPS-induced damage in the developing rat brain [150]. A possible explanation for these apparently contradictory observations may be that modulation of the cellular response to oxidative stress by Igf1 is cell-dependent [151]. Contrary to what has been observed in neurons [152], astrocyte-specific overexpression of IGF-1 protects hippocampal neurons [153].

Neuroprotection can also be elicited through OSM (oncostatin M), a cytokine with anti-inflammatory activities. In response to IL-1β or TNF-α, astrocytes produce prostaglandin E2, which then induces *Osm* expression in microglia [154]. Upon binding to a complex formed between its receptor, OSMR, and gp130 [155], OSM can in turn attenuate expression of IL-1β or TNF-α [156]. Therefore communications between microglia and astrocytes may account for the balance of protective and destructive actions by these cells. Although *Osm* expression levels were similar in 5XFAD and wild type mice, elevated expression of *Osmr* might increase the constitutive Osm activity detected in neurons, astrocytes and microglia [154]. Besides, the protective effect of Osm on neuronal cell death is mediated by the Jak/Stat3 signaling pathway [157]. In the 5XFAD brain, this neuroprotective effect may be attenuated through the OSM-induced upregulation of *Socs3*, a known inhibitor of Jak/Stat signaling [157].

Finally, GRN/PGRN, an AD marker [158], is involved in the modulation of the neuroinflammatory response. Supporting this role, microglia display increased *Grn/Pgrn* expression following a variety of acute and chronic insults to the central nervous system [159]. Grn/Pgrn can increase endocytosis of extracellular peptides such as Aβ and affects microglial proliferation, recruitment, differentiation, activation and phagocytosis [159]. Grn/Pgrn is a potent inhibitor of TNF-α and promotes the upregulation of anti-inflammatory cytokines such as Il-4, Il-10 and Il-5 [159]. Direct interaction of GRN/PGRN with the TNF receptor also blocks the pro-inflammatory actions of TNF-α [160]. However, GRN/PGRN can be cleaved by extracellular proteinases such as MMP-9, −12 and −14 to produce granulin/GRN peptides, which increase the expression of IL-1β, IL-8 and TNF-α [161,162]. Therefore, a balance between PGRN and its processed form GRN may determine the contributions of certain cell types or subtypes to neuroprotection or neuroinflammation and their impact on the 5XFAD pathophysiology.

### Limitations of the study

The aim of this study was to provide an overview of the main networks affected in 5XFAD brain, as revealed by intensive data mining on dysregulated genes observed in transcriptomic profiles. Such a global approach determines tendencies based on the design of networks rather than confirms individual gene modulation at the transcript or protein levels. Given the stringent criteria chosen to filter our data, most of the genes within the described networks are significantly modulated in both hippocampus and cortex from M4 to M9. Investigating the role of non-shared DEGs at given time points and analyzing specificities of each brain region is beyond the scope of this study, but deserves further investigation in order to elucidate the precise molecular mechanisms at play. Importantly, many DEGs and several signaling pathways, associated to AD, have been found to be misexpressed in the 5XFAD model by two independent studies [30,31].

Further understanding of the signaling pathways affected in this mouse model, with the aim to link it to human AD, would entail broadening the study to additional brain regions. The entorhinal cortex, for instance, is known to be the site of early neuron loss in human AD and has recently been shown to be affected by amyloid deposition, as early as 2 months of age in both female and male 5XFAD mice [163].It would therefore be of great interest to investigate the transcriptomic profile of this brain region in future studies.

The study would also benefit from a comparison of transcriptomic profiles between female and male mice. Female 5XFAD animals are more affected at the histological and behavioural levels than male mice [9,163], possibly as a consequence of decreased estrogen levels. Variations in estrogen levels can directly impact on transcriptomic profiles through modulation of the genome and signaling pathways. In the current study, IPA analysis revealed a large proportion of DEGs potentially regulated by estrogens and one of their receptors, ESR1 (data not shown). We also observed a dysregulated expression of genes directly linked to the effects of testosterone, reinforcing the idea that differential gene modulation during the time course of the disease could occur between male and female mice. Future studies should take hormonal impact into consideration.

Finally, based on the data presented here and in previous studies, there is no doubt that a fine understanding of pathogenesis necessitates deciphering early molecular events. As a result, it would be of great importance to enlarge the current study to a finely tuned time window that spans for M1 to M4, when histological markers start developing.

Despite such considerations, the data presented here offers, for the first time, the possibility to understand time-dependent variations in the inflammatory and immune pathways of the 5XFAD model. Altogether, these data confirm that this transgenic model, along with the generated dataset, is a valuable public resource for screening potential therapeutic molecules targeting dysregulated functions in AD.

## Materials and methods

### Animals

We used 5XFAD transgenic mice, which overexpress two transgenes bearing five mutations linked to familial AD: human *APP* (Swedish mutation K670N, M671L; Florida mutation I716V; London mutation V717I) and human *presenilin 1* (*PSEN1* M146L, L286V), under transcriptional control of the mouse Thy1 promoter. 5XFAD lines from the B6SJL genetic background were maintained by crossing hemizygous transgenic mice with B6SJL F1 breeders. These mice exhibit AD-related symptoms earlier than other animal models and amyloid deposition starts in the cortex and subiculum at 2 months of age [1]. Heterozygous female 5XFAD transgenic animals and wild type controls were obtained after breeding of progenitors purchased from the Jackson Laboratory. Genotyping was performed by PCR analysis of tail DNA in order to detect the human *APP* gene. Animal experiments were approved by the Ethics Committee of the Medical Faculty of Marseille and were carried out in accordance with the guidelines published in the European Communities Council Directive of November 24, 1986 (86/609/EEC). All efforts were made to reduce animal suffering and the number of mice.

### RNA isolation

Brain tissues were collected from wild type and transgenic 5XFAD mice (n = 3 per group). At different designated time points (beginning of M1, M4, M6 and M9), mice were anesthetized with isoflurane and sacrificed to extract brain tissue. Hippocampus and neocortex samples were dissected, snap-frozen in liquid nitrogen and stored at −80°C until use. Total RNA was then isolated from frozen hippocampi and cortices using RNeasy Mini kit (Qiagen, Courtaboeuf, France), according to the manufacturer’s instructions. RNA concentration was determined using a Nanodrop 2000 spectrophotometer (Thermo Scientific, ThermoFisher Scientific, Villebon sur Yvette, France) and RNA integrity assessed on an Agilent 2100 Bioanalyzer (Agilent Technologies, Les Ulis, France).

### Real-time quantitative PCR (qPCR)

Before performing microarray experiments, RNA samples extracted from the hippocampus of all animals (n = 3) in each group were tested with qPCR in order to quantify the expression of known markers of inflammation in 5XFAD mice. Total RNA (1 μg) was subjected to reverse transcription reaction to synthetize cDNA using oligo dT, RNase Out and M-MLV RT enzyme (Invitrogen, ThermoFisher Scientific, Villebon sur Yvette, France) according to the manufacturer’s instructions. Two genes, *Gfap* and *Aif1*, related to astrocytic and microglial activation respectively, and one housekeeping gene, *Gapdh*, were selected for pre-validation of samples.

Real-time qPCR experiments were carried out with the 7500 Fast Real-Time PCR system (Applied Biosystems, ThermoFisher Scientific, Villebon sur Yvette, France), using TaqMan® Fast Universal PCR Master Mix (2X) and the three TaqMan® Gene Expression Assays (Gfap, Mm01253033_m1; Aif1, Mm00479862_g1 and Gapdh, Mm99999915_g1). Experiments used 7.5 ng of previously prepared cDNA and samples were run in triplicates. Relative expression levels were determined according to the ΔΔCt method where the expression level of the mRNA of interest is given by 2^-ΔΔCT^ where ΔΔCT = ΔCT target mRNA − ΔCT reference mRNA (*Gapdh*) in the same sample. Results are reported in Table 4 and compared to microarray data for these two genes of interest.

**Table 4:** RT qPCR results for two genes known to be upregulated in 5XFAD mice: comparison with the microarray results

		**qPCR**							**Microarray**		
**Age**	**Gene**	**Wt 1**	**Wt 2**	**Wt 3**	**Tg 1**	**Tg 2**	**Tg 3**	**Fold change Tg/Wt**	**Wt**	**Tg**	**Fold change Tg/Wt**
M1	*Aif1*	1.3	0.9	0.8	1.3	1.3	0.9	**1.2**	2098.9	2010.3	**1.0**
	*Gfap*	1.5	0.9	0.7	1.3	1.4	0.8	**1.2**	6320.3	6827.6	**1.1**
M4	*Aif1*	1.1	1.1	0.9	2.8	1.7	1.3	**3.1**	2171.1	5335.4	**2.5**
	*Gfap*	0.9	1.3	0.9	5.5	6.1	5.1	**5.5**	5381.9	15329.9	**2.9**
M6	*Aif1*	0.9	1.0	1.2	2.8	1.7	1.3	**1.9**	2427.4	5530.1	**2.3**
	*Gfap*	1.1	1.0	0.9	3.9	5.6	4.2	**4.5**	5694.4	17759.7	**3.1**
M9	*Aif1*	1.4	0.9	0.8	2.6	2.0	2.3	**2.3**	2551.1	7422.3	**2.9**
	*Gfap*	1.5	0.8	0.9	5.0	7.0	5.9	**5.9**	5172.5	23062.3	**4.5**

Before performing the microarray experiment, RNA from each wild type (Wt) and each transgenic (Tg) (n = 3 for each group) animal was tested independently by RT-qPCR for two genes known to be upregulated in 5XFAD mice: *Aif1* and *Gfap.* This table compares results obtained by qPCR from each mouse with results obtained from the pooled RNA for all three animals in the microarray study. The qPCR and microarray gave similar fold changes, shown here in bold.

### Microarray assay

Following qPCR pre-validation of individual animals for each time point and condition, RNA samples were pooled (n = 3) for microarray hybridization. Sample amplification, labeling, and hybridization were performed in line with the Agilent one-color microarray-base analysis (low input quick amp labeling) protocol (Agilent Technologies). Briefly, total RNA was reverse-transcribed into cDNA using the T7 promoter primer. The reaction intending to synthesize cyanine-3-labeled cRNA from cDNA was performed in a solution containing dNTP mix, T7 RNA polymerase and cyanine 3-dCTP and then incubated at 40°C for 2 hours. Labeled cRNA was purified and fragmented before hybridization on Agilent 8×60k Mouse Gene Expression Arrays (Agilent Technologies, ref: G4852A), containing 62 975 oligonucleotide probes, at 65°C for 17 hours. Raw microarray signals were scanned and extracted using Agilent Feature Extraction Software (Agilent Technologies). AgiND R package was used for quality control and normalization. Quantile methods and a background correction were applied for data normalization. Microarray data are available in the ArrayExpress database [164] under accession number E-MTAB-1937.

### Microarray data analysis

Biological interpretation of the data was performed using two different programs. First, Ingenuity Pathway Analysis (IPA, Ingenuity Systems [165]) was used to identify biological functions from the lists of DEGs associated to transgenic animals. The main criteria to validate a differentially expressed gene was a fold change over 1.5 or under −1.5 when considering expression values in the transgenic group relative to the wild type control group. Upregulated and downregulated genes were analyzed in the same datasets to obtain the biologically relevant function categories. Right-tailed Fisher’s exact test was used to calculate a p-value determining the top statistically significant biological functions assigned to the data set.

Secondary analysis of the main metabolic pathways and their potential dysfunctions was performed using the Java/Perl software Predictsearch® (Laboratoire Genex [166]), which has been previously described [69,74]. This software characterizes the pathways and functional networks in which the selected genes found to be up- or down-regulated are involved. For this mechanistic analysis, only genes not differentially expressed across all time points in wild type animals (ratio between 0.85 and 1.2) but upregulated in transgenic animals (ratio over 1.5) were considered. Predictsearch was then used to generate functional networks based on the total number of differentially expressed genes in both cortex and hippocampus.

## Abbreviations

5XFAD: A strain of transgenic mice that carry mutant human *APP* and human *PSEN1* genes harbouring a total of five mutations linked to familial AD; AD: Alzheimer’s disease; APP: Amyloid precursor protein; Aβ: Beta-amyloid peptide; Aβ42: Beta-amyloid peptide 1-42; DEG: Differentially expressed gene; FAD: Familial Alzheimer’s disease; FC: Fold change; GTPases: A large family of hydrolase enzymes that can bind and hydrolyze guanosine triphosphate (GTP); IFN-γ: Interferon gamma; IPA: Ingenuity pathway analysis; ISG: Interferon stimulated gene; M1: One month; M4: Four months; M6: Six months; M9: Nine months; MHC: Major histocompatibility complex; NADPH: Reduced form of NADP+, *i.e.* nicotinamide adenine dinucleotide phosphate; NOX: NADPH oxidase complex; PSEN1: Presenilin-1; Thy1: Thymocyte antigen 1.

## Competing interests

The authors declare that they have no competing interest.

## Authors’ contributions

VL isolated the RNA, helped perform the microarray assay, analyzed the results and wrote the manuscript. KB bred and genotyped the mice, and carried out the qPCR analysis. BL performed the microarray assay. IV and SR participated in interpreting the data and writing the manuscript. PB analyzed the microarray data and wrote the manuscript. FF designed and coordinated the study, and helped to draft the manuscript. All authors read and approved the final manuscript.

## Supplementary Material

Additional file 1: Table S1 Lists of genes and associated fold changes commonly dysregulated at M1, M4, M6 and M9 in (A) cortex and (B) hippocampus. Click here for file

Additional file 2: Table S2 Common DEGs in cortex and hippocampus at M4, M6 and M9. Click here for file
