# Supplementary material for: Temporal gene profiling of the 5XFAD transgenic mouse model highlights the importance of microglial activation in Alzheimer’s disease
Source: Mol Neurodegener. 2014 Sep 11;9:33. doi: 10.1186/1750-1326-9-33 (PMC4237952; doi:10.1186/1750-1326-9-33)
Supplement: Additional file 1: Table S1 — Lists of genes and associated fold changes commonly dysregulated at M1, M4, M6 and M9 in (A) cortex and (B) hippocampus. [file 1750-1326-9-33-S1.pdf]

A

	CORTEX	FOLD CHANGE			
Gene Symbol	Entrez Gene Name	M1	M4	M6	M9
5730416F02Rik	capping protein (actin filament), gelsolin-like pseudogene	4,0	2,0	1,8	1,8
9930111J21Rik1/Gm12185	predicted gene 12185	2,9	2,2	-1,9	1,8
AQP1	aquaporin 1 (Colton blood group)	1,9	2,5	1,6	-2,5
CA8	carbonic anhydrase VIII	-1,9	-1,8	1,5	2,4
CLIC6	chloride intracellular channel 6	4,7	2,5	1,5	-1,5
Eci3	enoyl-Coenzyme A delta isomerase 3	-2,2	5,3	-1,5	1,7
FCGR2B	Fc fragment of IgG, low affinity IIb, receptor (CD32)	1,5	1,6	1,9	3,9
GABBR2	gamma-aminobutyric acid (GABA) B receptor, 2	-1,5	2,6	-1,7	1,8
Gm5878	predicted gene 5878	-2,0	2,4	-1,8	1,7
IFI16	interferon, gamma-inducible protein 16	-1,5	2,2	2,4	2,4
KCNE2	potassium voltage-gated channel, Isk-related family, member 2	4,7	3,7	1,5	-1,6
KIF12	kinesin family member 12	1,5	-1,5	-1,6	2,2
MED8	mediator complex subunit 8	-1,5	-1,6	2,9	-1,5
NEGR1	neuronal growth regulator 1	-1,5	1,5	-1,6	2,1
NKD2	naked cuticle homolog 2 (Drosophila)	-1,5	1,6	-1,9	2,1
OMP	olfactory marker protein	1,7	6,2	1,6	-2,7
PAPPA2	pappalysin 2	-1,5	-1,5	-1,5	-2,4
REC8	REC8 meiotic recombination protein	-1,9	-2,0	-2,4	-2,5
SLC39A4	solute carrier family 39 (zinc transporter), member 4	1,5	2,0	1,8	1,7
ST6GALNAC1	ST6 (alpha-N-acetyl-neuraminyl-2,3-beta-galactosyl-1,3)-N-acetylgalactosaminide alpha-2,6-sialyltransferase 1	-1,9	1,6	-1,5	7,1
TOX4	TOX high mobility group box family member 4	-1,7	4,3	-2,7	2,1
Vmn1r188 (includes others)	vomeronasal 1 receptor 217	1,7	-1,5	-1,6	2,1
Xlr3c (includes others)	X-linked lymphocyte-regulated 3C	1,9	-1,8	1,5	-2,3

B

HIPPOCAMPUS		FOLD CHANGE			
Gene Symbol	Entrez Gene Name	M1	M4	M6	M9
5430417L22Rik	RIKEN cDNA 5430417L22 gene	-1,522	-1,481	-1,658	2,638
A230065H16Rik	RIKEN cDNA A230065H16 gene	3,897	1,581	1,58	2,093
ACTA2	actin, alpha 2, smooth muscle, aorta	-3,502	-1,977	2,107	1,597
ADAMTS2	ADAM metallopeptidase with thrombospondin type 1 motif, 2	-1,881	-1,481	-1,466	1,554
ADI1	acireductone dioxygenase 1	1,463	1,509	1,61	1,541
AGA	aspartylglucosaminidase	-1,462	-1,461	2,153	1,724
AGPAT2	1-acylglycerol-3-phosphate O-acyltransferase 2	-1,564	1,48	1,624	1,945
AHNAK	AHNAK nucleoprotein	-1,547	1,876	1,568	1,785
ALDH1A2	aldehyde dehydrogenase 1 family, member A2	-1,987	-1,727	1,623	1,471
ANXA1	annexin A1	-1,646	-1,613	-1,612	1,598
Bst2	bone marrow stromal cell antigen 2	-2,404	1,592	2,675	3,918
CD48	CD48 molecule	1,659	2,454	3,457	3,741
CD300LF	CD300 molecule-like family member f	1,469	2,697	5,351	4,348
CDK5RAP1	CDK5 regulatory subunit associated protein 1	-1,642	-1,581	-1,505	-2,166
CNN1	calponin 1, basic, smooth muscle	-3,335	-2,036	1,873	1,806
CTSH	cathepsin H	-1,709	2,009	1,945	2,114
CXCL6	chemokine (C-X-C motif) ligand 6	1,663	2,735	1,566	5,658
CXCL16	chemokine (C-X-C motif) ligand 16	-1,614	1,586	3,121	2,452

EYA2	eyes absent homolog 2 (Drosophila)	-1,516	-1,508	-1,497	1,535
FAM46C	family with sequence similarity 46, member C	-1,964	3,689	2,398	5,958
FOXC2	forkhead box C2 (MFH-1, mesenchyme forkhead 1)	-1,472	-1,836	1,564	1,906
GBP6	guanylate binding protein family, member 6	3,941	1,776	2,298	3,719
Gm13194	predicted gene 13194	2,496	1,473	1,538	-2,027
GMPS	guanine monphosphate synthase	-1,551	-2,187	-1,668	-2,791
GPR182	G protein-coupled receptor 182	-1,547	-1,8	1,681	1,739
Gstm6	glutathione S-transferase, mu 6	-1,46	-1,49	-1,688	-1,715
Gvin1 (includes others)	GTPase, very large interferon inducible 1	-1,58	1,883	1,8	3,225
HAVCR2	hepatitis A virus cellular receptor 2	1,483	2,747	2,091	2,889
HHEX	hematopoietically expressed homeobox	-1,476	1,539	2,002	2,497
HLA-A	major histocompatibility complex, class I, A	-1,476	4,177	4,236	3,88
HLA-DRB5	major histocompatibility complex, class II, DR beta 5	-1,718	-1,466	8,292	3,382
Ifi27l2a/Ifi27l2b	interferon, alpha-inducible protein 27 like 2A	-2,387	1,645	2,623	4,203
ITIH2	inter-alpha-trypsin inhibitor heavy chain 2	-1,558	-1,599	1,525	1,643
KCNE2	potassium voltage-gated channel, Isk-related family, member 2	-9,745	1,687	-2,029	3,19
LDLR	low density lipoprotein receptor	1,565	1,722	1,598	2,025
LGALS3	lectin, galactoside-binding, soluble, 3	-1,947	1,788	2,726	5,693
LGALS3BP	lectin, galactoside-binding, soluble, 3 binding protein	-1,995	2,412	5,04	3,715
OXT	oxytocin/neurophysin I prepropeptide	2,988	-1,554	2,793	-7,806
PON3	paraoxonase 3	-2,14	2,116	1,981	3,608
PRKD3	protein kinase D3	-1,463	1,723	1,612	1,475
RABGAP1	RAB GTPase activating protein 1	3,128	1,634	1,47	3,272

REC8	REC8 meiotic recombination protein	-1,893	-1,949	-2,136	-2,051
SAMSN1	SAM domain, SH3 domain and nuclear localization signals 1	1,526	3,836	3,11	3,632
SCTR	secretin receptor	1,476	1,461	2,079	2,322
SHC4	SHC (Src homology 2 domain containing) family, member 4	3,572	1,606	3,286	3,329
SLC16A8	solute carrier family 16 (monocarboxylate transporter), member 8	-3,607	1,56	1,681	1,943
SLC37A2	solute carrier family 37 (glucose-6-phosphate transporter), member 2	-1,809	2,078	2,641	2,72
SLC6A12	solute carrier family 6 (neurotransmitter transporter), member 12	-2,277	-2,96	2,498	2,118
TLR1	toll-like receptor 1	-1,857	2,324	3,14	1,607
TLR2	toll-like receptor 2	-1,658	3,849	4,498	6,208
TNFRSF1A	tumor necrosis factor receptor superfamily, member 1A	-1,459	1,683	2,216	3,385
TOX4	TOX high mobility group box family member 4	-1,511	3,315	1,622	-1,547
UCP2	uncoupling protein 2 (mitochondrial, proton carrier)	-1,616	1,843	2,789	1,754
VIM	vimentin	-1,511	1,588	2,029	4,634
WFDC2	WAP four-disulfide core domain 2	-4,709	1,582	-1,748	2,139
