# Supplementary material for: Temporal gene profiling of the 5XFAD transgenic mouse model highlights the importance of microglial activation in Alzheimer’s disease
Source: Mol Neurodegener. 2014 Sep 11;9:33. doi: 10.1186/1750-1326-9-33 (PMC4237952; doi:10.1186/1750-1326-9-33)
Supplement: Additional file 2: Table S2 — Common DEGs in cortex and hippocampus at M4, M6 and M9. [file 1750-1326-9-33-S2.pdf]

		CORTEX			HIPPOCAMPUS		
**GeneName**	**Probe Name**	M4	M6	M9	M4	M6	M9
Dhx36	A_51_P148959	-1.60	-1.54	-1.75	-1.70	-1.45	-1.64
Gmps	A_55_P2185323	-1.86	-2.26	-2.08	-2.19	-1.67	-2.79
Rec8	A_51_P246677	-1.97	-2.38	-2.45	-1.95	-2.14	-2.05
5430435G22Rik	A_55_P2082989	2.00	1.65	4.41	2.88	2.61	4.12
5730416F02Rik	A_52_P110070	2.03	1.83	1.77	1.90	1.51	1.81
Abi3	A_51_P185906	2.32	2.75	3.23	2.65	2.95	3.09
Adora3	A_55_P1972605	1.66	1.89	1.47	2.19	1.95	2.60
Agpat2	A_55_P1970810	1.45	1.60	1.47	1.48	1.62	1.94
AI646519	A_55_P2359610	1.71	1.66	1.78	1.92	1.61	2.07
Aif1	A_51_P400543	2.15	2.44	2.18	2.46	2.28	2.91
Ang	A_51_P391159	1.53	1.45	1.74	1.98	2.27	1.49
Anxa3	A_55_P2056325	1.83	1.63	1.75	1.80	1.57	2.43
Apbb1ip	A_55_P1961009	1.83	1.79	2.11	2.72	2.71	2.91
Arhgap9	A_55_P2091551	2.00	2.52	2.14	2.37	2.70	3.27
Arpc1b	A_55_P2023076	1.78	2.07	2.57	1.88	1.55	3.86
Asb10	A_55_P2044710	2.25	1.52	2.80	2.64	4.01	2.32
Aspg	A_52_P338956	2.00	2.96	2.85	3.25	4.34	5.23
Atf3	A_52_P452689	2.24	2.62	2.08	1.81	3.84	2.45
B2m	A_51_P129012	1.74	1.63	2.54	1.78	2.45	2.45
B2m	A_51_P129012	1.66	1.66	2.74	1.64	2.75	2.53
Bcl3	A_55_P2066116	2.10	3.05	3.70	2.87	3.73	5.85
Bcl2a1c	A_52_P299771	2.78	2.48	3.21	2.43	4.43	3.64
Bst2	A_51_P169693	2.45	2.47	3.59	1.59	2.67	3.92
C1qa	A_51_P181451	2.65	3.25	3.98	3.02	3.84	4.68
C1qb	A_51_P351860	2.32	2.95	3.20	2.63	5.14	2.92
C1qc	A_51_P102789	2.83	2.00	5.67	3.03	4.58	4.20
C1qc	A_51_P102789	2.60	2.12	5.66	2.86	4.22	4.16
C1qc	A_51_P102789	2.56	2.07	5.52	2.82	4.47	4.27
C1qc	A_51_P102789	2.53	2.30	5.50	2.88	4.52	4.74
C1qc	A_51_P102789	2.60	2.21	5.66	2.85	4.13	4.63
C1qc	A_51_P102789	2.52	2.22	5.59	3.06	4.00	4.52
C1qc	A_51_P102789	2.55	2.32	5.58	2.91	4.12	4.51
C1qc	A_51_P102789	2.53	2.18	5.47	2.99	3.97	4.29
C1qc	A_51_P102789	2.58	1.97	5.75	2.97	5.40	4.12
C1qc	A_51_P102789	2.64	2.03	5.53	2.89	4.20	4.12
C3ar1	A_51_P282557	2.15	1.96	2.88	2.13	5.69	2.96
Capg	A_55_P1998631	2.57	2.15	3.66	2.78	2.71	3.85
Ccl4	A_51_P509573	8.97	7.31	9.91	12.71	10.67	11.32
Ccl6	A_51_P460954	12.44	9.41	13.37	13.66	10.81	9.74
Cd9	A_51_P320852	1.79	1.83	2.41	1.69	1.59	2.58
Cd14	A_51_P172853	2.74	3.20	3.82	2.57	3.63	3.30
Cd37	A_55_P2118570	2.31	2.09	3.18	2.16	2.71	2.74
Cd37	A_55_P2174490	1.98	1.96	2.90	1.90	4.28	2.09
Cd48	A_55_P2185605	2.21	2.74	2.46	2.45	3.43	3.74

Cd48	A_55_P2132549	1.86	2.37	3.00	2.10	3.46	2.79
Cd52	A_51_P151126	5.00	5.76	9.37	6.84	6.34	9.90
Cd68	A_55_P1961943	5.21	3.01	10.46	5.78	6.19	7.20
Cd84	A_55_P2054445	3.10	3.08	2.79	2.89	2.88	4.31
Cd86	A_55_P1971951	1.78	2.31	2.50	2.51	3.05	3.51
Cd300lf	A_55_P2081164	2.34	4.71	3.00	2.51	5.35	3.80
Cd300lf	A_52_P422494	2.27	3.84	2.72	2.70	4.80	3.18
Cebpa	A_52_P168567	2.09	1.66	2.66	1.90	2.08	1.87
Ch25h	A_55_P2008987	2.58	2.61	4.10	3.68	3.65	4.68
Clec7a	A_51_P246653	12.10	34.95	30.86	15.93	32.21	32.47
Col16a1	A_55_P2124751	1.21	1.95	1.36	2.03	1.56	2.09
Col16a1	A_55_P2056739	1.63	2.18	1.79	2.07	2.07	1.88
Csf1	A_51_P195506	1.58	1.63	1.86	1.44	1.69	2.16
Csf1	A_51_P195506	1.47	1.46	1.93	1.48	1.56	2.01
Csf1	A_51_P195506	1.54	1.44	1.72	1.42	1.62	1.99
Csf1	A_51_P195506	1.48	1.62	1.95	1.56	1.39	2.17
Csf1	A_51_P195506	1.55	1.61	1.97	1.70	1.40	2.22
Csf1	A_51_P195506	1.40	1.50	1.83	1.38	1.56	2.02
Csf1	A_51_P195506	1.44	1.73	1.82	1.35	1.53	2.50
Csf1	A_51_P195506	1.47	1.54	1.76	1.49	1.50	2.09
Csf1	A_51_P195506	1.46	1.63	1.81	1.51	1.42	2.24
Csf1	A_51_P195506	1.52	1.55	1.87	1.48	1.48	2.22
Csf1r	A_52_P602091	2.17	2.47	1.68	1.84	2.40	2.07
Csf1r	A_55_P2025078	1.83	1.88	1.89	2.19	1.70	2.51
Csf1r	A_55_P2025073	1.42	1.40	1.26	1.30	1.71	1.03
Csf2rb	A_52_P52618	2.12	2.07	2.26	2.02	2.41	3.06
Cst7	A_51_P137419	74.71	63.46	140.15	100.48	118.36	145.84
Ctsa	A_55_P1955841	1.50	1.69	1.74	1.75	1.20	2.07
Ctsc	A_52_P195839	1.95	1.94	1.96	1.77	2.07	2.40
Ctsd	A_51_P384629	3.04	3.02	3.79	3.23	3.08	3.48
Ctsd	A_65_P13209	2.97	4.44	2.41	2.83	3.72	3.14
Ctsh	A_52_P51078	1.85	1.52	1.66	2.01	1.94	2.11
Ctss	A_55_P2024888	3.03	3.32	2.88	3.40	3.75	4.04
Ctsz	A_51_P256202	2.71	3.02	3.85	3.02	3.78	4.33
Cx3cr1	A_52_P99810	1.99	1.01	3.01	2.10	2.64	2.00
Cx3cr1	A_55_P2007964	1.98	1.64	1.61	2.16	2.03	2.30
Cxcl10	A_55_P2016462	2.86	3.42	3.02	2.50	3.77	5.35
Cyba	A_55_P1979341	3.08	2.72	4.63	2.69	2.44	4.68
Cyba	A_51_P131800	2.33	1.57	3.73	2.38	2.37	3.05
Cyth4	A_55_P2113210	2.03	2.13	2.57	1.79	1.47	2.42
Emr1	A_55_P2018934	1.52	1.26	1.62	1.61	2.02	1.85
Emr1	A_55_P2144386	1.61	1.57	1.40	1.93	1.68	2.37
F420015M19Rik	A_55_P2041584	1.63	-1.55	3.13	2.15	1.47	2.03
Fam167b	A_52_P326657	1.52	1.49	1.89	2.20	1.97	1.89
Fam46c	A_51_P447976	2.92	2.77	4.88	3.69	2.40	5.96
Fblim1	A_55_P2038757	1.47	1.51	2.61	1.59	4.68	2.78

Fcer1g	A_51_P405476	3.28	2.42	5.04	3.57	3.98	3.83
Fcgr2b	A_51_P130095	1.60	1.67	3.82	1.97	3.38	4.16
Fcgr2b	A_51_P130095	1.58	1.69	3.46	2.35	3.07	5.25
Fcgr2b	A_51_P130095	1.52	1.68	3.48	2.32	2.41	5.58
Fcgr2b	A_51_P130095	1.42	1.70	3.90	2.50	2.14	6.25
Fcgr2b	A_51_P130095	1.52	1.79	3.73	1.95	3.29	5.97
Fcgr2b	A_51_P130095	1.54	1.51	3.93	2.19	2.57	4.97
Fcgr2b	A_51_P130095	1.54	1.85	3.56	1.76	3.96	5.48
Fcgr2b	A_51_P130095	1.46	1.67	3.00	2.41	3.34	4.86
Fcgr2b	A_51_P130095	1.55	1.59	3.05	2.22	3.03	5.27
Fcgr2b	A_51_P130095	1.44	1.66	3.69	2.72	2.80	4.72
Fes	A_52_P365011	2.05	1.91	1.90	1.88	2.13	1.96
Fes	A_52_P635338	1.96	2.38	2.22	2.66	1.95	3.13
Fyb	A_55_P2006869	1.75	2.15	1.83	1.63	1.54	2.75
Gal3st2	A_55_P2026547	1.76	1.89	1.86	2.01	1.48	2.65
Gfap	A_55_P2157245	3.37	6.08	4.36	2.20	2.97	5.37
Gfap	A_52_P52303	5.16	8.37	5.01	2.85	3.12	4.46
Gfap	A_55_P2157250	8.08	8.58	11.81	3.99	7.98	3.50
Glipr1	A_51_P371051	1.98	1.77	1.54	1.47	1.66	1.56
Gm5637	A_55_P2166555	2.14	2.07	2.60	2.27	2.07	2.92
Gngt2	A_55_P2151986	1.83	1.77	3.02	2.62	2.32	3.46
Gpnmb	A_51_P438967	1.76	3.44	2.37	1.97	5.68	3.70
Gpr34	A_55_P2039684	1.98	1.92	2.12	2.36	2.05	2.63
Gpr65	A_51_P108459	2.30	2.92	2.67	2.28	2.75	3.50
Gpr65	A_51_P108459	2.34	2.54	2.76	1.86	2.10	3.19
Gpr65	A_51_P108459	2.74	1.37	2.75	2.51	2.95	2.01
Gpr65	A_51_P108459	2.89	2.96	2.89	2.87	2.84	4.53
Gpr65	A_51_P108459	2.54	3.34	2.64	2.44	3.13	4.62
Gpr65	A_51_P108459	2.49	3.17	2.48	2.11	1.92	3.97
Gpr65	A_51_P108459	2.69	2.92	2.64	2.81	-1.38	3.49
Gpr65	A_51_P108459	2.44	3.05	2.61	2.96	2.88	3.51
Gpr65	A_51_P108459	2.13	2.72	2.41	1.84	2.70	3.09
Gpr65	A_51_P108459	2.61	2.79	2.44	2.67	2.75	3.61
Gpr84	A_55_P2044932	2.78	2.85	3.78	3.10	2.22	5.77
Gpsm3	A_55_P1957633	1.79	1.49	2.47	1.83	1.90	2.10
Grn	A_51_P192800	2.55	2.33	2.64	2.20	3.03	2.47
Gsdmd	A_51_P172231	1.25	1.62	1.58	1.38	2.04	1.60
Gsdmd	A_51_P172231	1.45	1.71	1.54	1.26	2.16	1.51
Gsdmd	A_51_P172231	1.32	1.66	1.54	1.44	1.76	1.68
Gsdmd	A_51_P172231	1.63	1.74	1.62	1.45	2.21	1.82
Gsdmd	A_51_P172231	1.48	1.88	1.61	1.34	2.21	1.83
Gsdmd	A_51_P172231	1.43	1.59	1.67	1.42	2.20	1.87
Gsdmd	A_51_P172231	1.43	1.75	1.64	1.37	2.84	1.84
Gsdmd	A_51_P172231	1.54	1.77	1.39	1.62	1.73	1.85
Gsdmd	A_51_P172231	1.51	1.92	1.50	1.52	2.24	1.57
Gsdmd	A_51_P172231	1.47	1.66	1.45	1.33	1.86	1.66

Gusb	A_51_P211491	2.49	3.17	3.24	3.05	3.61	3.89
Gusb	A_51_P211491	2.92	2.90	2.85	2.93	3.07	3.64
Gusb	A_51_P211491	2.67	3.41	3.30	3.09	3.30	4.19
Gusb	A_51_P211491	2.55	3.46	3.16	3.14	2.76	3.82
Gusb	A_51_P211491	2.80	3.38	3.33	2.88	2.67	4.20
Gusb	A_51_P211491	2.72	3.04	3.11	2.83	2.85	3.54
Gusb	A_51_P211491	2.62	3.56	3.14	3.09	2.96	4.59
Gusb	A_51_P211491	2.77	3.42	3.17	2.96	3.26	4.13
Gusb	A_52_P160936	1.33	1.45	1.54	1.63	1.32	2.00
Gusb	A_51_P211491	2.66	2.95	3.33	3.12	3.42	3.80
Gusb	A_51_P211491	2.73	3.41	3.11	2.98	2.98	3.90
H2-M2	A_51_P191463	1.54	1.31	1.51	1.44	3.51	-1.08
H2-M2	A_51_P191463	1.11	1.70	1.47	1.33	2.70	1.41
H2-M2	A_51_P191463	1.24	1.42	1.55	1.46	2.60	1.63
H2-M2	A_51_P191463	1.54	1.29	1.53	1.55	2.42	1.60
H2-M2	A_51_P191463	1.48	1.41	1.28	1.37	2.56	1.51
H2-M2	A_51_P191463	1.55	1.21	1.59	1.37	2.76	1.49
H2-M2	A_51_P191463	1.35	1.35	1.57	1.25	2.62	1.34
H2-M2	A_51_P191463	1.40	1.42	1.43	1.53	3.10	1.33
H2-M2	A_51_P191463	1.37	1.24	1.34	1.32	2.57	1.35
H2-M2	A_51_P191463	1.05	1.29	1.80	1.37	2.69	1.36
H2-Q8	A_55_P1978465	2.03	3.34	2.68	1.51	5.38	3.35
Haao	A_55_P2074035	2.04	1.51	1.86	1.50	1.57	1.50
Havcr2	A_55_P1965659	2.14	2.41	2.75	2.75	2.09	2.89
Hcls1	A_51_P297679	2.08	2.50	2.33	2.29	2.27	3.37
Hcst	A_55_P1984886	1.85	1.84	3.68	2.90	2.26	3.69
Hexa	A_51_P282667	1.90	2.01	2.31	1.72	2.33	2.39
Hexb	A_51_P453111	2.49	2.04	3.20	2.81	2.04	3.28
Hk2	A_51_P204080	1.68	1.81	2.06	1.52	2.53	2.40
Hmha1	A_55_P2002103	2.01	2.58	2.36	2.51	2.09	3.16
Hspb6	A_55_P2039289	1.55	1.45	2.08	1.60	1.58	1.74
Hvcn1	A_55_P2025008	2.76	3.22	3.72	2.83	2.92	3.95
Hvcn1	A_55_P1960053	2.72	3.93	4.07	2.87	3.36	4.51
Igf1	A_55_P2085979	1.42	-1.24	1.04	1.06	1.30	-1.39
Igf1	A_55_P2085984	2.70	2.42	3.72	3.22	2.84	5.41
Igf1	A_55_P2085984	2.53	2.13	5.02	3.85	2.86	5.35
Igf1	A_55_P2031636	1.17	1.01	1.16	1.03	1.58	1.50
Igf1	A_55_P2031631	2.98	2.48	4.57	2.96	2.77	5.27
Igf1	A_55_P2031631	2.59	1.97	5.09	3.06	2.99	4.22
Igsf6	A_51_P258372	1.64	1.81	1.85	1.95	1.76	2.55
Ikzf1	A_66_P124139	1.33	1.35	1.29	1.76	1.71	1.79
Ikzf1	A_55_P2023607	1.47	1.55	1.76	2.09	1.75	2.56
Il3ra	A_55_P2008181	1.79	2.21	2.12	1.85	1.82	2.12
Inpp5d	A_51_P337125	1.67	1.87	2.00	2.11	1.99	3.47
Irf5	A_51_P346668	2.06	2.03	2.06	2.30	1.77	2.10
Irf8	A_52_P354823	3.17	3.20	4.29	3.70	3.96	4.24

Itgax	A_51_P303424	16.29	13.43	19.61	16.11	14.83	23.20
Itgb2	A_51_P262208	2.47	2.65	3.39	2.35	2.38	2.87
Itgb5	A_51_P327796	2.01	1.62	2.68	1.87	1.73	2.45
Kcne2	A_55_P1979242	3.69	1.49	-1.63	1.69	-2.03	3.19
Klhl6	A_51_P433615	2.07	2.69	1.56	1.66	2.11	2.75
Krt85	A_55_P1999364	2.62	3.10	5.40	2.58	3.36	3.78
Lag3	A_51_P264825	2.84	2.27	3.64	2.73	4.53	3.21
Lair1	A_55_P1991214	1.63	1.38	2.36	2.13	2.13	2.46
Lat2	A_51_P242930	1.72	1.94	2.20	1.86	2.39	1.97
Lcp1	A_55_P2066613	1.41	1.21	2.52	2.62	2.35	3.11
Lcp1	A_52_P56751	1.74	1.88	1.63	1.91	2.09	2.40
Lgals3	A_55_P2171116	1.91	2.96	4.24	1.79	2.73	5.69
Lsp1	A_55_P1957413	1.85	2.10	2.34	2.26	2.45	3.28
Ly86	A_51_P465350	3.55	4.47	4.02	4.15	4.41	4.71
Lyl1	A_51_P143190	1.51	1.66	1.94	1.61	1.71	2.11
Man2b1	A_51_P336161	2.20	2.43	2.17	2.16	2.27	2.53
Mlph	A_66_P132222	1.61	2.29	1.72	1.93	2.48	1.59
Mlxipl	A_55_P2119957	2.22	2.52	2.22	2.56	2.74	2.45
Mpeg1	A_51_P390538	5.58	5.52	6.06	7.74	7.35	8.19
Myo1f	A_66_P112305	2.32	2.79	3.30	3.64	2.68	4.98
Myo1f	A_55_P2124273	2.59	3.59	3.20	2.95	4.26	3.19
Naglu	A_52_P504361	2.07	2.06	2.17	1.66	2.24	2.03
Naip2	A_55_P1984243	2.20	2.80	2.37	2.12	2.21	3.19
Naprt1	A_51_P414115	1.64	1.75	1.76	1.54	1.48	1.57
Ncf2	A_55_P2184189	1.77	2.09	2.71	2.51	3.28	3.31
Ncf4	A_51_P377452	2.09	2.09	2.11	2.36	1.76	3.12
Nckap1l	A_51_P119429	1.95	2.20	2.11	2.43	1.66	3.22
Nckap1l	A_51_P119429	1.89	2.12	2.16	2.27	1.72	2.66
Nckap1l	A_51_P119429	1.83	1.82	2.03	2.31	1.61	2.68
Nckap1l	A_51_P119429	1.93	2.08	2.11	2.64	1.55	2.68
Nckap1l	A_51_P119429	1.79	1.93	1.96	2.60	1.93	2.79
Nckap1l	A_51_P119429	1.70	2.18	1.88	2.31	1.63	2.83
Nckap1l	A_51_P119429	1.85	1.94	2.13	2.38	1.96	2.75
Nckap1l	A_51_P119429	1.86	2.10	1.86	2.22	2.27	2.85
Nckap1l	A_51_P119429	1.89	2.17	2.02	2.57	1.85	2.94
Nckap1l	A_51_P119429	1.71	1.98	1.89	2.39	1.80	2.76
Npc2	A_51_P347961	1.85	2.27	1.96	1.63	1.99	2.51
Olfml3	A_51_P191782	2.24	1.92	1.87	2.00	1.90	1.62
Osmr	A_51_P319460	1.65	2.71	3.06	2.47	3.05	5.29
P2ry6	A_51_P105124	2.45	1.61	1.96	1.70	1.71	2.44
P2ry6	A_51_P105124	1.70	1.96	2.03	2.15	1.93	2.38
P2ry6	A_51_P105124	1.87	2.06	2.09	1.96	1.82	2.43
P2ry6	A_51_P105124	1.89	1.80	2.21	1.80	1.97	2.55
P2ry6	A_51_P105124	1.65	1.80	2.17	2.08	1.99	2.42
P2ry6	A_51_P105124	1.78	1.71	1.94	2.03	2.18	2.33
P2ry6	A_51_P105124	1.74	1.66	2.05	1.99	1.74	2.43

P2ry6	A_51_P105124	1.57	1.84	2.15	1.99	2.02	2.44
P2ry6	A_51_P105124	1.76	1.97	2.15	2.06	1.90	2.52
P2ry6	A_51_P105124	1.77	1.74	2.22	2.17	1.75	2.59
Pdcd1	A_51_P285736	2.82	2.43	4.02	3.33	2.22	3.48
Plcg2	A_51_P279163	1.68	1.92	1.89	1.61	1.96	2.38
Pld4	A_52_P591153	1.97	2.32	1.62	2.12	2.69	1.59
Plek	A_55_P1966194	2.15	2.76	2.63	2.99	3.14	4.18
Pon3	A_51_P256665	2.12	2.16	2.82	2.12	1.98	3.61
Psmb8	A_51_P345367	2.27	2.17	2.56	1.96	3.58	2.67
Ptpn6	A_52_P114722	2.60	3.15	3.47	2.99	2.76	3.73
Ptpn18	A_55_P2069306	2.03	2.05	3.28	2.42	2.27	4.07
Ptprc	A_55_P1990324	2.05	1.66	2.05	2.00	2.14	2.48
Pycard	A_51_P438821	1.95	1.90	2.35	2.63	1.89	2.60
Rac2	A_51_P121891	2.06	1.83	2.43	2.47	3.52	2.40
Rac2	A_51_P121891	2.15	2.07	2.35	1.98	3.24	2.45
Rac2	A_51_P121891	2.08	2.12	2.46	1.98	2.52	2.58
Rac2	A_51_P121891	2.27	2.33	2.50	2.39	3.02	2.74
Rac2	A_51_P121891	2.05	2.02	2.45	2.24	2.70	2.51
Rac2	A_51_P121891	2.07	2.45	2.74	2.15	2.78	2.64
Rac2	A_51_P121891	2.24	2.15	2.38	2.08	3.16	2.38
Rac2	A_51_P121891	2.10	2.11	2.35	2.61	3.45	2.26
Rac2	A_51_P121891	1.94	2.43	2.39	2.15	2.56	2.60
Rac2	A_51_P121891	2.09	2.23	2.44	2.24	3.07	2.53
Rasal3	A_52_P16873	1.84	2.54	2.16	2.22	1.78	2.26
Renbp	A_55_P2013357	1.67	1.67	2.29	1.84	2.14	2.07
Runx1	A_51_P206585	1.64	1.51	1.71	1.35	1.04	2.02
Runx1	A_55_P2183438	1.14	1.14	1.48	1.52	1.79	1.67
Samsn1	A_55_P2010271	2.32	2.28	3.11	3.84	3.11	3.63
Selplg	A_55_P1976212	1.64	1.55	2.07	1.78	2.37	1.47
Selplg	A_51_P131358	2.13	1.91	2.05	2.01	1.95	1.81
Serpinf2	A_55_P1956497	1.90	2.32	3.41	2.60	4.81	7.43
Sh3bp2	A_55_P2042823	1.75	2.03	2.60	1.83	1.89	3.00
Siglec5	A_55_P2043277	2.10	2.27	1.95	1.81	1.54	2.71
Slamf9	A_51_P246066	4.57	4.97	4.47	4.22	3.55	4.60
Slc11a1	A_51_P186476	3.60	5.16	4.09	5.26	8.50	4.61
Slc14a1	A_51_P312336	1.92	2.54	3.06	1.67	1.92	2.81
Slc15a3	A_52_P467389	3.81	4.84	4.26	4.27	9.07	4.38
Slc16a3	A_55_P2067505	1.85	2.81	2.07	1.90	2.11	2.25
Slc16a3	A_55_P1959425	1.83	2.50	2.12	1.70	2.13	1.90
Slc37a2	A_55_P2350617	1.64	1.94	2.05	2.08	2.02	2.72
Slc7a7	A_51_P472249	1.67	1.62	2.11	2.35	1.81	2.15
Slco2b1	A_55_P2079009	1.71	1.86	1.83	1.97	1.94	2.47
Slco2b1	A_55_P2030155	1.46	1.75	1.94	1.63	2.25	2.10
Snx20	A_51_P309158	1.65	1.60	1.65	1.99	1.67	1.71
Sp110	A_55_P2152566	1.75	2.17	1.95	1.90	2.16	2.85
Stard4	A_52_P384574	1.63	1.47	1.87	1.89	1.57	2.23

Syngr1	A_51_P270733	3.03	2.22	4.17	3.06	3.82	3.18
Syt10	A_51_P318830	7.28	-3.34	2.47	3.89	2.15	-2.29
Tbxas1	A_51_P448664	2.52	2.85	3.17	3.92	3.83	3.15
Tcirg1	A_51_P241861	1.51	1.75	1.86	1.68	2.06	1.46
Tgfbr2	A_55_P2107288	1.30	1.68	2.45	1.70	1.44	2.19
Tgfbr2	A_51_P450573	1.91	2.06	2.27	2.20	1.98	3.68
Tgif1	A_55_P2092526	1.71	1.77	2.28	2.15	1.74	3.65
Timp2	A_65_P12392	1.84	1.76	2.62	1.95	2.10	1.99
Tlr1	A_51_P495581	2.30	1.72	1.56	2.32	3.14	1.61
Tlr2	A_51_P452629	2.71	3.59	3.33	3.25	3.58	5.12
Tlr2	A_51_P452629	2.86	3.40	3.57	3.20	2.72	4.94
Tlr2	A_51_P452629	2.60	3.86	3.20	3.85	3.44	4.69
Tlr2	A_51_P452629	2.87	4.38	3.56	3.81	3.57	6.09
Tlr2	A_51_P452629	2.75	4.21	3.33	3.38	2.79	6.21
Tlr2	A_51_P452629	2.65	4.22	3.03	3.45	2.16	4.86
Tlr2	A_51_P452629	2.65	4.15	3.44	3.34	3.62	5.15
Tlr2	A_51_P452629	2.51	3.63	3.35	3.61	4.22	5.47
Tlr2	A_51_P452629	2.89	3.76	3.36	3.47	3.68	5.74
Tlr2	A_51_P452629	2.59	3.08	3.12	2.97	4.50	4.40
Tlr7	A_55_P2050932	1.34	1.41	1.48	1.81	1.43	1.41
Tlr7	A_55_P2129047	1.56	1.80	2.19	2.11	4.14	2.68
Tlr12	A_66_P114333	2.04	2.00	2.15	2.29	2.80	3.01
Tlr13	A_55_P2048279	1.91	1.89	2.32	2.29	1.96	2.91
Tnfaip8l2	A_51_P150678	1.66	2.15	2.35	1.77	1.49	2.67
Tnfrsf1a	A_52_P192426	1.36	1.69	1.98	1.38	1.60	2.73
Tnfrsf1a	A_52_P192426	1.42	1.83	2.15	1.59	1.90	2.92
Tnfrsf1a	A_52_P192426	1.45	2.01	2.32	1.68	1.65	3.38
Tnfrsf1a	A_52_P192426	1.46	1.73	2.18	1.48	1.37	3.19
Tnfrsf1a	A_52_P192426	1.53	1.82	2.24	1.68	1.66	3.04
Tnfrsf1a	A_52_P192426	1.35	2.03	2.34	1.56	2.22	3.30
Tnfrsf1a	A_52_P192426	1.48	1.97	2.24	1.47	1.74	3.09
Tnfrsf1a	A_55_P2051099	1.31	1.45	1.91	1.41	2.07	1.29
Tnfrsf1a	A_52_P192426	1.61	1.68	2.09	1.61	1.81	2.89
Tnfrsf1a	A_52_P192426	1.41	1.89	2.14	1.42	1.74	2.77
Tnfrsf1a	A_52_P192426	1.49	1.60	2.16	1.38	1.65	2.77
Tnfsf8	A_55_P2179463	1.87	1.56	2.44	2.58	1.48	2.92
Tnni2	A_55_P2040893	2.40	2.00	3.49	2.96	2.56	2.75
Tox4	A_55_P2121618	4.35	-2.72	2.12	3.32	1.62	-1.55
Trem2	A_55_P1962937	7.05	4.23	10.78	9.00	5.53	11.92
Tubb6	A_51_P421140	1.62	1.67	1.54	1.78	1.74	3.02
Tyrobp	A_51_P261517	5.82	6.44	6.40	5.74	5.11	7.24
Ucp2	A_51_P297105	1.91	1.60	2.58	1.84	2.79	1.75
Ucp3	A_66_P108247	2.18	1.84	2.29	1.69	2.68	2.00
Unc93b1	A_52_P507382	2.42	1.78	2.50	2.20	1.98	2.42
Vav1	A_51_P154780	1.66	1.92	1.77	1.98	1.48	2.04
Vav1	A_55_P2036567	2.02	1.96	2.64	2.59	1.75	3.71

Vim	A_51_P392687	1.58	1.64	2.69	1.59	2.03	4.63
Was	A_51_P327121	1.67	1.57	1.64	2.20	2.76	1.79
Wdfy4	A_52_P199614	1.65	1.58	1.87	2.02	2.07	3.00
